# Medicinal use of non-prescribed cannabis: a cross-sectional survey on patterns of use, motives for use, and treatment access in the Netherlands

**DOI:** 10.1186/s42238-025-00355-y

**Published:** 2025-12-02

**Authors:** Lisa Strada, Simone Korteling, Mark Vergeer, Pieter Oomen

**Affiliations:** Trimbos Institute, Da Costakade 45, Utrecht, 3521 VS The Netherlands

**Keywords:** Medical cannabis, Non-prescribed use, Self-medication, Cannabis policy, Harm reduction, Access to treatment

## Abstract

**Background:**

Despite the Netherlands having one of the world’s oldest medical cannabis programs, the majority of people who use cannabis for medicinal purposes continue to rely on non-prescribed sources. This study investigates patterns of use, motives for use, perceived effectiveness, and barriers to accessing prescribed cannabis among individuals self-medicating with non-prescribed cannabis.

**Methods:**

A cross-sectional online survey was conducted between January and April 2023, using convenience sampling primarily via social media. Participants (*N* = 1059) were adults (18 years or older) residing in the Netherlands who self-reported current use of non-prescribed cannabis-based products to manage physical or mental health symptoms.

**Results:**

Cannabis was used to manage a wide range of conditions, most commonly chronic pain, sleep disorders, depression, and ADHD/ADD, with three out of four participants reporting use for multiple conditions. Most participants obtained cannabis from coffeeshops, although one in four also reported home cultivation as a source. Participants typically smoked cannabis with tobacco, reported (near-)daily use for therapeutic purposes, and indicated a monthly expenditure of €100. The majority was not aware of the THC and CBD content of their products. Perceived effectiveness was rated as high, and more than half of those with a history of prescription medication use reported substituting cannabis for these medications. Only a minority of participants had ever used, or were currently using, prescribed cannabis. Commonly cited barriers included perceived lower quality, higher cost, and lower ease of access compared with non-prescribed cannabis.

**Conclusions:**

The widespread use of non-prescribed cannabis for medicinal purposes in the Netherlands reflects both unmet health needs and barriers within the regulated medical cannabis system. Risky use practices – such as smoking cannabis with tobacco and using products without knowing their cannabinoid content – raise public health concerns. The findings highlight the need for harm reduction strategies and policies that better align medical cannabis regulation with patients’ real-world behaviours and care needs.

**Supplementary Information:**

The online version contains supplementary material available at 10.1186/s42238-025-00355-y.

## Background

Cannabis has been legally available by prescription for medicinal use in the Netherlands since 2003 [1], while cannabis for recreational use through coffeeshops^1^ has been tolerated since the 1970 s [2]. Despite having one of the world’s long-standing medical cannabis program, most individuals who use cannabis for therapeutic purposes continue to rely on non-prescribed sources [3]. A 2020 survey of a representative sample of the Dutch general population found that among those using cannabis medicinally, 92.7% used only non-prescribed cannabis, 4.7% used only prescribed cannabis, and 2.6% used both [3]. These figures suggest that nearly half a million adults in the Netherlands use non-prescribed cannabis for medicinal purposes. In contrast, only around 7300 patients were prescribed cannabis in 2023 [4]. This disparity raises important questions about who the non-prescribed users are, what health conditions they use cannabis for, and why they self-medicate despite the legal availability of cannabis through the healthcare system.

The use of non-prescribed cannabis for medicinal purposes raises significant public health concerns. Cannabis products obtained from coffeeshops or informal networks are not subject to consistent quality control, and the cannabinoid content is often unknown or labelled inaccurately [5]. The products may also contain harmful contaminants such as pesticides, heavy metals, or microbial agents, posing additional health risks [6–9]. The absence of professional medical oversight exacerbates these risks. Healthcare professionals can play a vital role in advising patients on dosing, monitoring treatment outcomes and side effects, and managing potential interactions with other medications [1]. In the Netherlands, however, only a small number of physicians are likely equipped to provide such guidance, as medical cannabis is not covered in standard medical training. To date, no studies have examined Dutch physicians’ knowledge or perspectives on medical cannabis, but anecdotal evidence suggests that patients often struggle to find doctors who are willing or feel equipped to prescribe it.

Cannabis may be prescribed in the Netherlands when regular treatment options and medications are not effective enough or cause too many side effects [1]. There is no predefined list of eligible medical conditions, which means prescribing decisions are largely left to the discretion of physicians [1]. Five cannabis strains with varying concentrations of tetrahydrocannabinol (THC) and cannabidiol (CBD) are available. These strains can be dispensed in dry form or processed by specialized pharmacies into sublingual oils and topical lotions tailored to individual patient needs. Any licensed physician is authorized to prescribe cannabis; [1] however, it is generally not reimbursed by health insurers [10]. While no national registry of prescribed cannabis patients exists, a recent study suggests that cannabis is most commonly prescribed for chronic pain [11]. In assessing access pathways, it is useful to compare the Dutch model to those in other countries. Germany and Australia, for example, have introduced fast-track access through telehealth clinics, which have come under scrutiny for potentially catering to recreational users. By contrast, patients in the Netherlands are typically required to try different conventional treatments under the supervision of a physician before cannabis may be considered. As a result, inappropriate prescribing of cannabis is less likely to pose a serious concern in the Dutch context.

Although several international studies have examined the characteristics and motivations of individuals using non-prescribed cannabis for medicinal purposes [12–15], no such research has been conducted in the Dutch context. The Netherlands presents a unique context, where prescribed medical cannabis and tolerated recreational use have coexisted for over two decades, offering a distinct opportunity to investigate patterns of non-prescribed medicinal cannabis use. Findings from other countries, such as Germany, Belgium, the United Kingdom, and Australia, show that the primary reasons for medicinal cannabis use are chronic pain, mental health conditions and sleep problems [12–15]. Herbal cannabis is the most commonly used form, and smoking – often mixed with tobacco – remains the predominant route of administration [12–15]. Medicinal cannabis users are also more likely to be unemployed or unable to work [13, 15]. In Australia and Germany, where medical cannabis is legally available, perceived barriers include high cost, reluctance among physicians to prescribe cannabis, and stigma regarding cannabis use [12, 14]. A German study found that many participants continued to rely on black market or home-grown cannabis despite having physician-diagnosed conditions. Interestingly, more than half of the participants had never requested a prescription for cannabis, and did not feel the need to do so, as they considered illicit sources sufficiently reliable, affordable, and of good quality. Nevertheless, respondents indicated a preference for prescribed medical cannabis if these barriers were addressed [14]. 

## Methods

### Aim

The aim of the study is to examine patterns of use, motives for use, perceived effectiveness, and access to prescribed cannabis among individuals using non-prescribed cannabis for medicinal purposes. By examining the characteristics, behaviours, and barriers faced by this group, the study seeks to inform policies and interventions that promote safer use and improved access to prescribed cannabis.

### Study design and data collection

This study employed a cross-sectional online survey design using a convenience sample of individuals who self-reported current use of cannabis for medicinal purposes. Medicinal use was defined as the use of cannabis-based products to alleviate self-reported physical or mental health symptoms. The online questionnaire was programmed using Jambo Software (version 3.2) and was accessible via a public link for three months from 19 January to 19 April 2023. Participants were recruited through multiple channels, including targeted advertisements on Facebook, announcements in the monthly newsletter and website of the Trimbos Institute, and printed flyers distributed at ten coffeeshops across the Netherlands. Additionally, the survey gained traction through online platforms that reposted the survey link, including general news websites, cannabis-focused media, health platforms, and advocacy groups for patients and cannabis consumers. Participation was anonymous. Upon completing the questionnaire, participants had the option to enter a lottery for one of ten €200 prizes. The study was granted an exemption from ethics from the Medical-Ethical Review Committee METC NedMec (reference 22–912/DB).

### Eligibility criteria

Participants were eligible to take part in the survey if they were 18 years or older, resided in the Netherlands, had sufficient proficiency in Dutch, and self-reported current use of non-prescribed cannabis-based products to manage physical or mental health symptoms. Individuals using both prescribed and non-prescribed cannabis were included in the study, while those using only prescribed cannabis or only commercial CBD products were excluded. Over-the-counter CBD products were excluded because they contain substantially lower CBD concentrations than CBD prescription medications [5] and because evidence supporting their efficacy is lacking [16]. 

### Questionnaire

The questionnaire was informed by previous surveys on medicinal cannabis use [12, 17, 18] and developed through an iterative process involving researchers who are experts in the field and people with lived experience of using cannabis for medicinal purposes. These individuals were identified through the researchers’ professional networks. Feedback was collected with the aim of addressing thematic gaps in previous questionnaires and improving content validity. For instance, to better reflect real-world use, participants could report the use of multiple cannabis products rather than just one. The questionnaire was pilot-tested with five medicinal cannabis users, demonstrating good comprehensibility, acceptability, and relevance. Final revisions were made based on pilot feedback. The final questionnaire covered eight domains: (1) Source of cannabis, (2) Patterns of use, (3) Cannabis composition, (4) Conditions and symptoms managed with cannabis, (5) Patient-reported outcomes, (6) Prescription medication use, (7) Access to prescribed cannabis, and (8) Experiences with prescribed cannabis. The average completion time was 14 min. The original Dutch version of the questionnaire, along with a non-validated English translation, is available in the Supplementary Material.

### Statistical analyses

Descriptive statistics were calculated using R software (version 4.4.1). No imputation was performed for missing data. The number of responses varied across certain items due to automated questionnaire routing, whereby follow-up questions were shown only to participants who gave relevant answers to preceding items. Most items used predefined response categories. Responses to open-ended questions were analysed using open coding to identify emergent themes.

Several plausibility checks were performed, including assessments of internal consistency across related questions. Responses to optional open-ended items were reviewed to ensure that most participants provided at least one meaningful written entry, indicating human input. In addition, participants’ email addresses, which were collected for follow-up studies, were checked for duplicates and screened to ensure they appeared valid and most likely human-generated rather than bot-generated.

## Results

### Participant sample

Of the 1633 individuals who provided informed consent, 521 were excluded for not meeting the eligibility criteria. The most common reasons were not using cannabis products for physical or mental health symptoms (*n* = 320) and exclusive use of commercially available CBD products (*n* = 103). Additional exclusions (*n* = 98) were due to being under 18 years of age, not residing in the Netherlands, or using only prescribed cannabis. During data cleaning, a further 53 participants were excluded for the following reasons: incomplete demographic data (*n* = 3), non-serious responses (*n* = 2), duplicate entries (*n* = 11; second entry was removed), exclusive use of prescribed cannabis (*n* = 2), and survey discontinuation before completion (*n* = 35). The demographic characteristics of excluded individuals were similar to those of the final sample. The final sample comprised 1059 participants.

Most participants reported learning about the study via Facebook (67.5%). Other recruitment sources included other social media platforms (14.8%), cannabis-related websites (5.9%), non-cannabis related websites (4.3%), personal network (3.7%), cannabis social clubs (non-profit organizations in which cannabis is grown and distributed to its members, 0.4%), coffeeshops (0.4%), and other sources^2^ (2.9%).

### Participant characteristics

Participant characteristics are reported in Table 1. The sample was predominantly male (59.4%) and had a mean age of 45.1 years (SD 14.9). Individuals aged 50–59 years represented the largest age group (31.6%). Most participants had completed upper secondary or vocational secondary education (41.5%) and most reported being unfit for work or disabled (39.1%).

**Table 1:** Sociodemographic characteristics of the study sample (*N* = 1059)

Sociodemographic characteristics	% or Mean (SD, range)
Gender	
- Male	59.4
- Female	38.1
- Other	1.3
- Prefer not to say	1.2
Age	
- Mean (SD, range)	45.1 (± 14.9, 18–82)
- 18–29	18.9
- 30–39	18.6
- 40–49	19.8
- 50–59	31.6
- 60+	11.0
Highest education level attained	
- Primary education	4.4
- Lower or pre-vocational secondary education	23.9
- Upper or vocational secondary education	41.5
- Bachelor’s or equivalent	19.4
- Master’s or Doctorate	4.7
- Not applicable/Don’t know	6.1
Employment status	
- Unfit for work/disabled	39.1
- Full-time work	25.2
- Part-time work	15.0
- Retired	9.7
- Unemployed	5.9
- Student	5.0

Note: Dutch equivalents of education levels, from lowest to highest: Basisonderwijs; VMBO, MBO1, praktijkonderwijs, onderbouw HAVO/VWO; HAVO, VWO, MBO; HBO, WO Bachelor; WO Master, Doctoraat

### 1. Source of cannabis

The vast majority of participants (94.5%) reported exclusive use of non-prescribed cannabis for medicinal purposes, while a small group (5.5%) used both prescribed and non-prescribed cannabis. These proportions closely align with findings from a general population survey in the Netherlands, in which approximately 95% of medicinal cannabis users relied on non-prescribed cannabis [3].

**Fig. 1:**
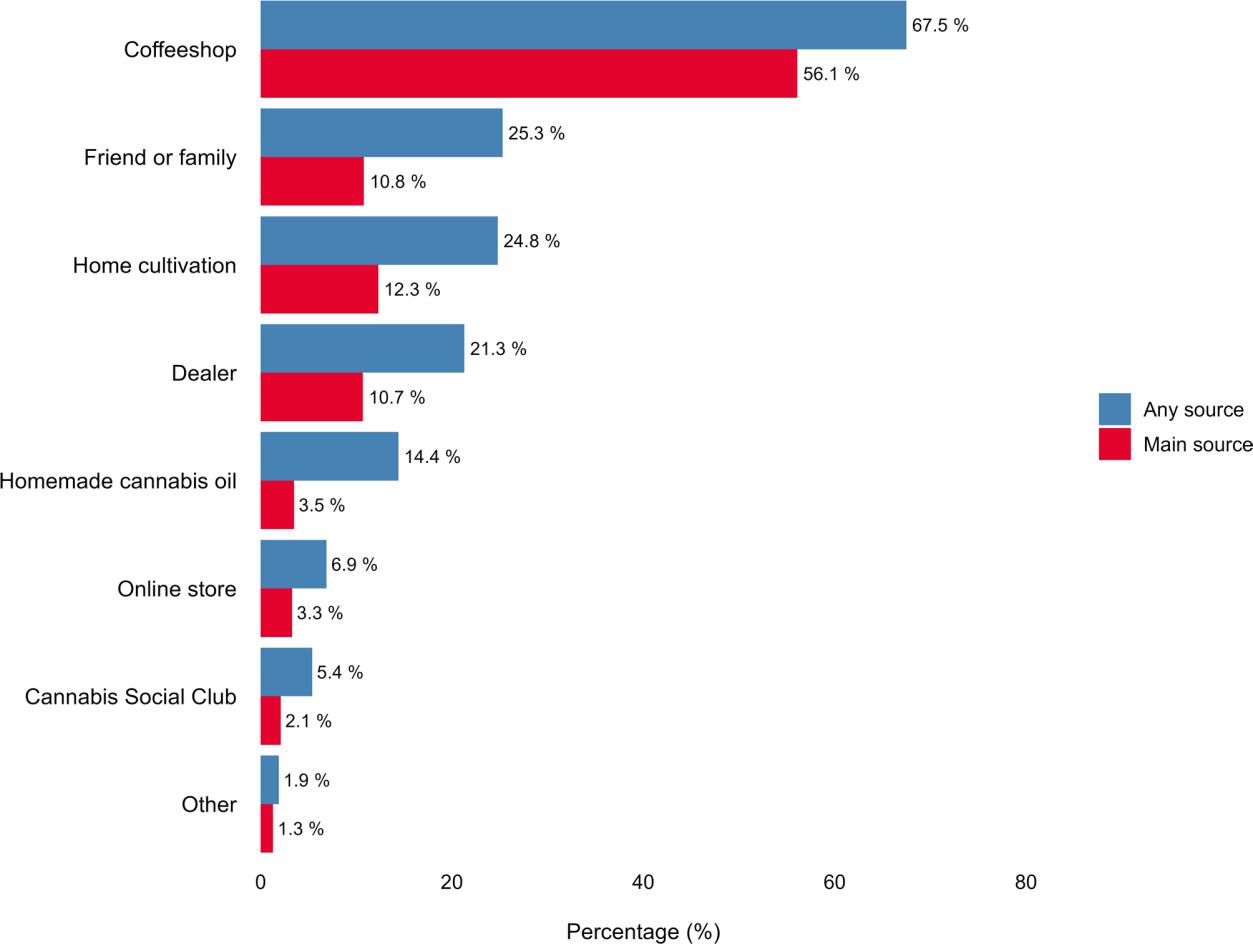
Any and main source of non-prescribed cannabis for medicinal purposes (*N* = 1059)

Participants were presented with a structured list and asked to select up to six sources from which they obtained non-prescribed cannabis for medicinal purposes (‘any source’) and to identify the source they used the most (‘main source’; Fig. 1). Coffeeshops were the most commonly reported source (67.5%), followed by friends or family (25.3%), home cultivation (24.8%), and dealers (21.3%). When asked to specify their main source, more than half of participants (56.1%) reported coffeeshops. About half of participants (53.1%) reported obtaining cannabis from a single source, 31.6% from two sources, and 15.3% from three or more sources.

### 2. Patterns of use

#### Product form

Participants were asked to indicate, from a structured list, up to six forms of cannabis they used for medicinal purposes (‘any form’) and to identify the form they used most frequently (‘main form’; Table 2). Herbal cannabis was the most commonly reported form, cited by 81.2% of participants, followed by sublingual/oral cannabis oil (37.2%) and cannabis resin (33.2%). When asked to specify their main form, 71.1% selected herbal cannabis. Nearly half of participants (48.6%) reported using a single form of cannabis, 34.7% used two forms, and 16.7% used three or more forms.

**Table 2:** Patterns of non-prescribed medicinal cannabis use: product form, route of administration, and concurrent recreational use (*N* = 1059)

	%	%
Product form	Any	Main
- Herbal cannabis	81.2	71.1
- Sublingual/oral cannabis oil	37.2	17.7
- Cannabis resin	33.2	8.2
- Cannabis topicals	11.6	0.9
- Cannabis oil to vaporize	7.3	0.3
- Other	4.9	1.8
Route of administration	Any	Main
- Smoke cannabis with tobacco	63.8	59.6
- Sublingual cannabis oil/tincture	31.8	16.2
- Smoke cannabis pure	27.9	11.4
- Oral consumption in food/drink	21.3	3.6
- Vaporize the cannabis flower	19.7	6.7
- Topical use of cream/lotion/salve	10.2	0.7
- Vaporize cannabis oil/extract	4.9	0.2
- Other	2.1	1.6
Recreational use	Past	Current
- Never	26.3	29.8
- Rarely	19.6	21.9
- Sometimes	27.9	32.5
- Often	26.3	15.8

#### Route of administration

Participants were asked to select, from a structured list, up to seven routes of administration (ROAs) used for medicinal cannabis (‘any ROA’) and to identify the ROA they used most frequently (‘main ROA’; Table 2). The most commonly reported ROA was smoking cannabis with tobacco (63.8%), followed by the sublingual use of cannabis oil (31.8%), smoking cannabis pure (i.e., without tobacco, 27.9%), and oral consumption via food or drink (21.3%). When asked to specify their main ROA, 59.6% of participants reported smoking cannabis with tobacco. Over half of participants (53.5%) reported using one ROA, 24.4% used two ROAs, and 22.1% used three or more ROAs.

Participants were also asked to indicate the reason for choosing their main ROA. They could select up to three reasons from a structured list. Across all ROAs, the most frequently reported reasons were ease of dosing (55.3%), quick onset of effects (50.0%), and ease of use (36.0%). Distinct patterns emerged when reasons were examined by ROA. Participants who mainly smoked cannabis (with or without tobacco; *n* = 752) favoured this method for its quick onset of effects (56.0%), ease of dosing (54.3%), and ease of use (31.6%). Those who mainly vaporized cannabis (plant material or oil/extract; *n* = 73) reported doing so because of the reduced harm to the lungs (64.4%), ease of dosing (54.8%), and quick onset of effects (53.4%). Participants who mainly consumed cannabis orally (as sublingual oil or in food/drink; *n* = 210) preferred this method due to the ease of dosing (60.0%), ease of use (53.3%), and reduced harm to the lungs (40.5%).

#### Recreational use

Participants were asked about their recreational cannabis use before and after initiating medicinal use (Table 2). The majority (73.7%) reported recreational use prior to using cannabis for medicinal purposes, and 70.2% reported current recreational use. The frequency of recreational use declined following the onset of medicinal use. Frequent recreational use decreased from 26.3% in the past to 15.8% in the present.

#### Frequency and duration of use

**Fig. 2:**
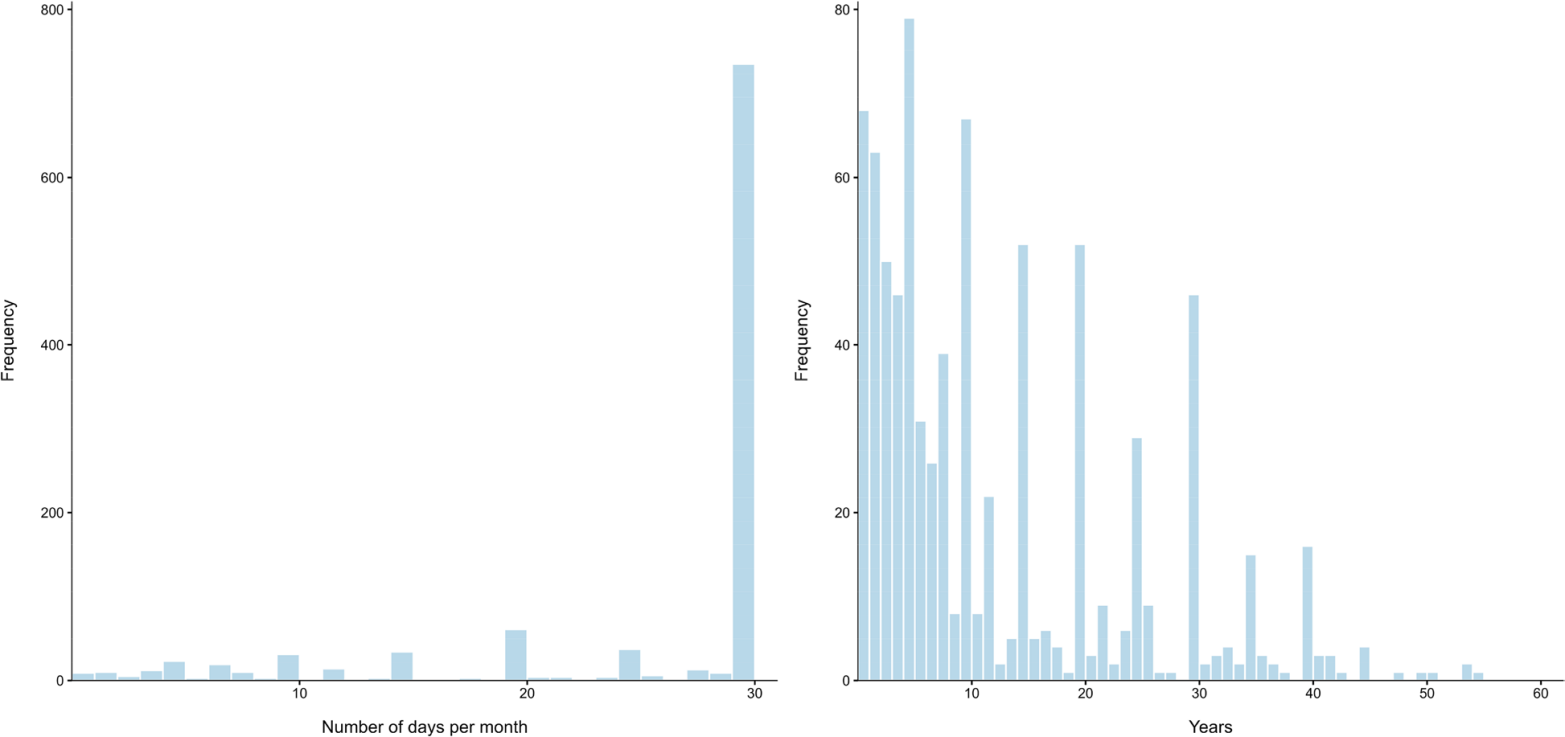
Frequency (*N* = 1059) and duration (*n* = 805) of medicinal use of non-prescribed cannabis

Most participants reported (near-)daily use^3^ of non-prescribed cannabis for medicinal purposes, with 82.8% using it on 20–30 of the past 30 days (Table 3). The median frequency of use was 30 days (IQR = 25, 30), with a mean of 25 ± 8.3 days (Fig. 2).

The duration of non-prescribed medicinal cannabis use varied considerably across the sample. A total of 110 participants did not complete this variable and a further 144 participants were excluded from analysis, because they provided implausible values. Implausible values were defined as cases where the difference between age and duration of medicinal cannabis use implied initiation of medicinal use before age 16. The median duration of medicinal cannabis use for the remaining 805 participants was 9 years (IQR = 4, 20), with a mean of 12.7 ± 11.5 years (Fig. 2). Nearly one-third of participants (32.9%) had used non-prescribed cannabis medicinally for fewer than 5 years, whereas 11.9% reported use for more than 30 years (Table 3).

**Table 3:** Frequency, duration, and monthly expenditure of non-prescribed medicinal cannabis use

	*n*	% or Median
Frequency of use (days in past 30 days)	1059	
- Median (IQR; Mean ± SD, Range)		30 (25, 30; 25 ± 8.3, 1–30)
- 1–3 days		2.3
- 4–9 days		6.6
- 10–19 days		8.3
- 20–30 days		82.8
Duration of use (years)	805	
- Median (IQR; Mean ± SD, Range)		9 (4, 20; 12.7 ± 11.5, 0.3–55)
- Less than 5 years		32.9
- 5–10 years		19.6
- 10–15 years		11.1
- 15–20 years		9.5
- 20–25 years		7.3
- 25–30 years		7.8
- 30 years or more		11.9
Monthly expenditure (€)	631	
- Median (IQR; Mean, Range)		100 (50, 200; 158.5, 1.5–1000)
- €1–100		41.4
- €101–200		24.6
- €201–300		14.6
- €301–400		10.1
- €401–1000		9.3

#### Monthly expenditure

**Fig. 3:**
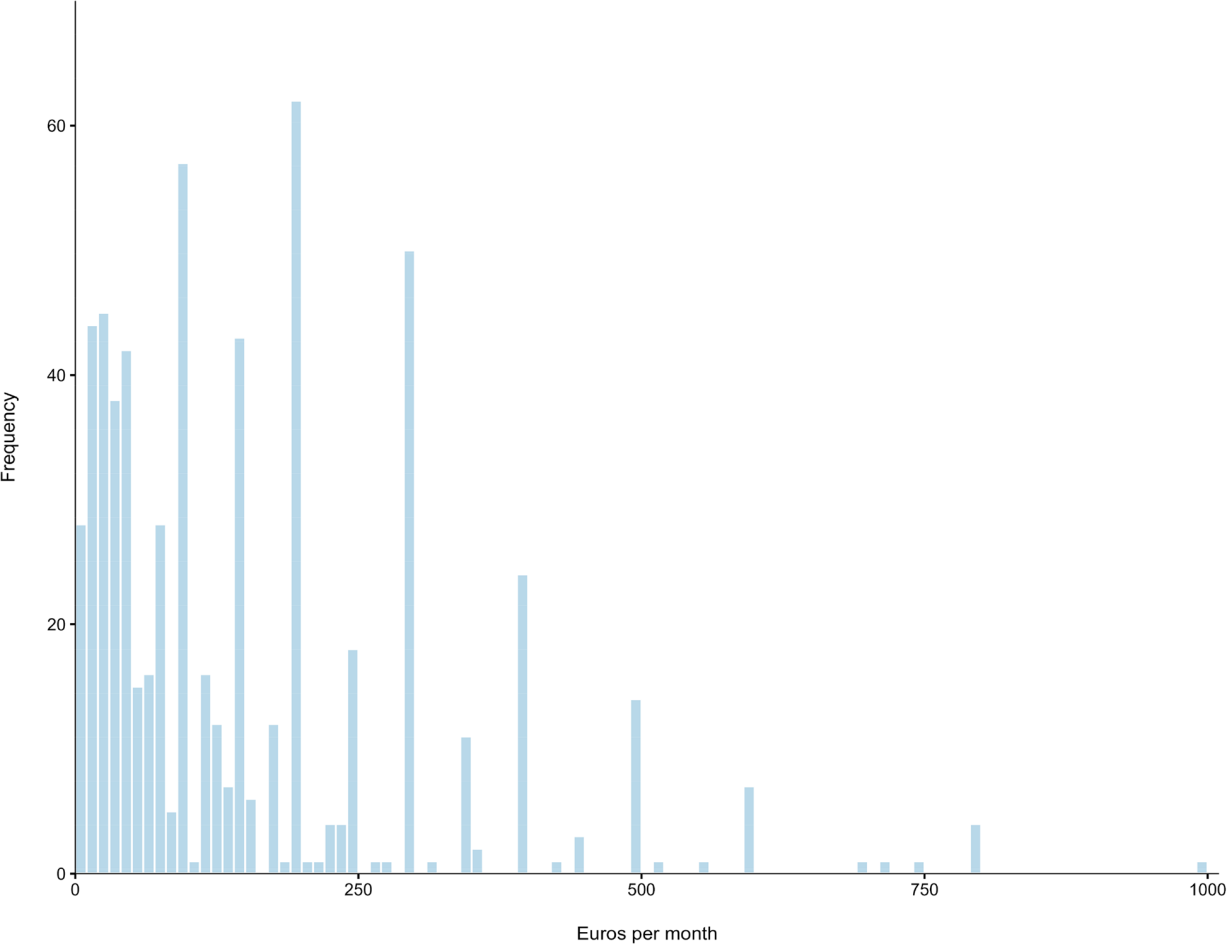
Self-reported monthly cost of non-prescribed cannabis used for medicinal purposes (*n* = 631)

Participants were asked how much money they spent per month on non-prescribed cannabis for medicinal purposes. While 13.8% reported not paying for their cannabis and 26.6% preferred not to say, 59.6% indicated that they paid for their cannabis. Among those who reported paying (*n* = 631), the median monthly expenditure was €100 (IQR €50–200, range €1.50–1000) (Fig. 3). Most participants (41.4%) reported spending up to €100 a month, while 9.3% spent between €400 and €1000 a month (Table 3).

To examine whether there was an association between payment status and source of cannabis, a series of chi-square tests of independence were conducted. Results indicate a strong associations between payment status and source of cannabis. Participants who did not pay for cannabis were significantly more likely to grow it themselves (64% vs. 19%) or make their own oil (45% vs. 9%). Conversely, participants who paid for cannabis were more likely to obtain it from coffeeshops (75% vs. 19%) or dealers (24% vs. 6%) (all *p* <.001). No significant differences were observed for cannabis social clubs, friends/family, online shops, or other sources.

### 3. Cannabis composition

#### Perceived THC and CBD content

Participants were asked to estimate both the level and concentration of THC and CBD in the non-prescribed cannabis product they most frequently used for medicinal purposes. Detailed information on cannabinoid concentrations is often unavailable in the Netherlands, as coffeeshops are not legally required to provide this information^4^. Instead, products are typically described in qualitative terms such as ‘high’, ‘medium’ or ‘low’ THC or CBD content. Therefore, participants were first asked to classify their cannabis using these descriptors (Table 4). Most reported using cannabis with medium or high THC levels, and medium or low CBD levels. A considerable proportion of participants reported not knowing the THC level (15.4%) or CBD level (29.6%) of their product.

**Table 4:** Percentage of participants estimating THC and CBD levels of their non-prescribed cannabis (*N* = 1059)

	THC level	CBD level
High	36.4%	11.6%
Medium	42.8%	35.8%
Low	5.4%	23.0%
Don’t know	15.4%	29.6%

Participants were subsequently asked, via an open-ended question, to estimate the THC and CBD concentrations of the cannabis product they used most frequently. If they were unsure, they could skip the question. To assess the plausibility of the estimates, responses were compared with data from the Dutch Coffeeshop Monitor [5], which tracks THC and CBD concentrations in cannabis sold in coffeeshops in the Netherlands. More than half of participants (55.8%) did not provide an estimate of THC/CBD concentration, 35.3% reported plausible values, and 8.9% reported implausible values (see footnote in Table 5 for plausibility criteria). The relatively high proportion of implausible responses (about one-fifth of all given estimates) raises concerns about the reliability of the self-reported cannabinoid concentrations. Nevertheless, plausible responses were analysed, although findings should be interpreted with caution. Median estimated THC concentrations were 20.0% for herbal cannabis, 22.5% for resin cannabis, and 15.0% for sublingual cannabis oil (Table 5). Median estimated CBD concentrations were below 6% across all three product types.

**Table 5:** Estimated THC and CBD concentrations in non-prescribed cannabis used for medicinal purposes (*n* = 374)

	THC concentration		CBD concentration	
Cannabis form	Median	Range	Median	Range
Herbal cannabis (*n* = 272)	20.0%	0–40%	2.25%	0–40%
Resin cannabis (*n* = 18)	22.5%	0.5–49%	5.0%	0–14%
Sublingual oil (*n* = 84)	15.0%	0–40%	5.25%	0–30%

Note. These findings are based on plausible values. Plausible THC and CBD values were defined based on data from the Dutch Coffeeshop Monitor [5], allowing a 10% margin of error. Maximum combined concentration thresholds of THC and CBD in cannabis products were set at 40% for herbal cannabis, 80% for resin cannabis, and 55% for sublingual cannabis. Since THC and CBD levels are interdependent (meaning their combined concentrations cannot exceed certain limits), self-reported values were considered implausible if their sum exceeded the set thresholds. If one of the two values was missing, both were classified as missing

Note. Medians are reported instead of means, because THC and CBD concentrations in herbal and resin cannabis in the Netherlands are not normally distributed [5]

#### Source of information on THC and CBD content

Participants were asked how they determined the THC and CBD content of their non-prescribed medicinal cannabis. Responses across the sample were as follows: 39.4% reported not knowing the cannabinoid content, 18.1% relied on personal estimation, 14.3% used product labels, 11.0% obtained information from coffeeshop staff, 7.7% relied on details provided by the person who sold or supplied the cannabis, and 9.4% selected ‘other’ sources. Among the latter, some participants reported having their cannabis tested privately.

### 4. Conditions and symptoms managed with cannabis

Participants were asked whether they used cannabis to manage medical conditions diagnosed by a physician or to alleviate non-specific symptoms without a formal diagnosis. The vast majority (*n* = 1001, 94.5%) reported using cannabis for physician-diagnosed medical conditions, while 5.5% (*n* = 58) used it for non-specific symptoms. Participants subsequently selected the specific medical conditions or the symptoms for which they used cannabis from structured lists^5^.

**Fig. 4:**
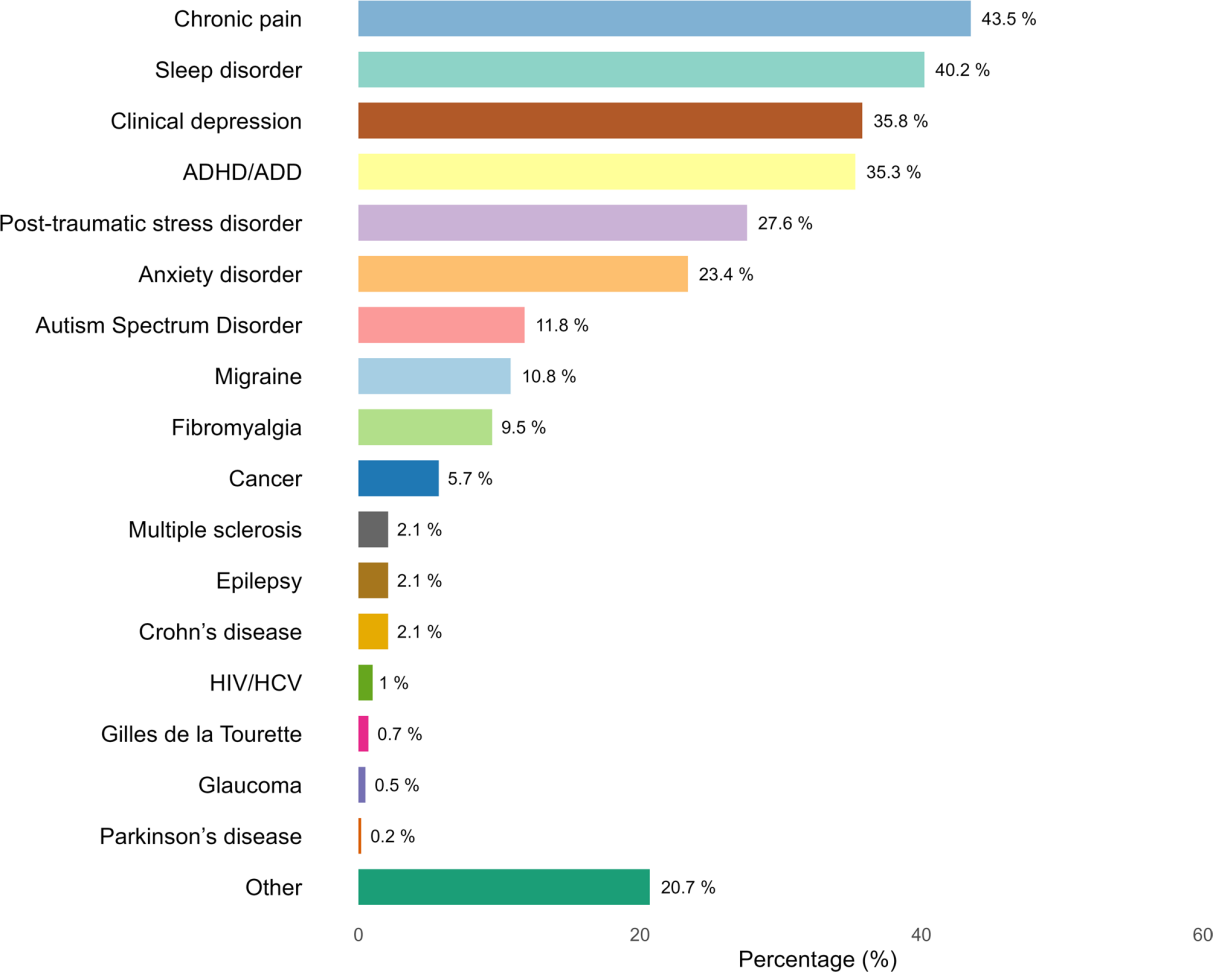
Conditions reported as reasons for using cannabis medicinally (*n* = 1001)

The most frequently reported medical conditions were chronic pain (*n* = 435, 43.5%), sleep disorders (*n* = 402, 40.2%), clinical depression (*n* = 358, 35.8%), ADHD/ADD (*n* = 353, 35.3%), post-traumatic stress disorder (PTSD, *n* = 276, 27.6%), and anxiety disorder (*n* = 234, 23.4%). Other reported conditions are presented in Fig. 4. Responses entered in the free-text field for ‘Other conditions’^6^ covered a diverse range of health issues, including rheumatic diseases, spinal disorders, gastrointestinal conditions, neuropathic pain, dermatological and gynaecological conditions, as well as connective tissue disorders (e.g., Ehlers-Danlos syndrome), neurological disorders (e.g., Restless Legs Syndrome), and various psychiatric disorders (e.g., Borderline Personality Disorder). Use of cannabis for multiple conditions was common, reflecting high rates of comorbidity in the sample. Specifically, 25.4% reported using cannabis for one condition, 25.8% for two conditions, 21.6% for three conditions, and 27.2% for four or more conditions.

Among participants who reported using cannabis for non-specific symptoms (*n* = 58), the most frequently cited issues were sleep problems (*n* = 26, 44.8%), stress or nervousness (*n* = 23, 39.7%), depressive symptoms (*n* = 16, 27.6%), chronic pain (*n* = 13, 22.4%), and muscle aches or cramps (*n* = 11, 19.0%). These largely mirror the most commonly reported medical conditions in the other subsample, including chronic pain, sleep disorders, and clinical depression. Most participants reported managing multiple non-specific symptoms with cannabis: 43.1% used cannabis for one symptom, 13.8% for two symptoms, 15.5% for three symptoms, and 27.6% for four or more symptoms.

### 5. Patient-reported outcomes

#### Perceived effectiveness

Participants were asked to report the extent to which non-prescribed cannabis improved their symptoms and quality of life (QOL). Responses were provided on a scale from 1 (no improvement) to 10 (complete improvement), with an additional option to indicate if cannabis worsened their symptoms or QOL. Nearly all participants reported some degree of improvement. The mean self-reported score was 7.88 (*n* = 1048) for symptom improvement and 7.95 (*n* = 1037) for QOL improvement. Because a score of 1 indicated ‘no improvement’, the scale functioned as a 9-point measure of change. Scores were therefore recoded to a 100-point scale to better reflect the degree of improvement. On this adjusted scale, the mean scores corresponded to an 87.6% improvement in symptoms and an 88.3% improvement in QOL. A small minority of participants reported that cannabis worsened their symptoms (*n* = 11, 1.0%) and their QOL (*n* = 16, 1.5%).

#### Positive effects on wellbeing and functioning

The majority of participants (96.7%) reported positive effects of cannabis beyond symptom relief. Using a structured list, participants selected all positive effects that applied to them. The most commonly reported benefits were improved sleep (75.8%) and an enhanced ability to relax (69.8%). Other responses are shown in Table 6.

**Table 6:** Positive effects of medicinal cannabis use on wellbeing and functioning (*n* = 1024)

Positive effects	%
Improved sleep	75.8
Improved ability to relax	69.8
Reduced depression	44.5
Reduced anxiety	37.8
Improved appetite	37.2
Improved concentration	33.5
Improved ability to move	32.6
Improved social interactions	30.4
Improved motivation to do things	27.7
Improved ability to work	19.9
Other	4.4

#### User concerns

Nearly half of participants (48.7%) reported financial concerns related to the medicinal use of cannabis, with 26.4% indicating they worried ‘a bit’, 15.7% ‘somewhat’, and 6.6% ‘very much.’ The remaining 51.3% reported no financial worries. Among those without financial concerns (*n* = 543), 23.8% obtained their cannabis free of charge. Participants who were unemployed or unfit for work were significantly more likely to report financial concerns than those with other employment statuses (Wilcoxon rank-sum test, *p* <.001). Specifically, only 39.9% of participants who were unemployed or unable to work reported no financial worries, compared to 59.9% of participants with other employment statuses.

Participants were also asked to indicate, from a pre-defined list, any additional concerns related to their medicinal use of non-prescribed cannabis. The most frequently reported concerns were stigma associated with cannabis use (43.0%) and potential contaminants in non-prescribed cannabis (42.5%). Other concerns included the illegal status of cannabis (37.8%), potential health risks (36.2%), unstable supply (33.9%), and the risk of addiction (23.7%).

#### User preferences

Participants were asked to identify, from a structured list, up to five factors they considered important when choosing a cannabis product for medicinal use. The most frequently cited factors were high THC content (47.0%), organic cultivation (41.3%), and pleasant taste (33.8%). Other preferences are detailed in Table 7.

**Table 7:** Factors considered important when choosing a cannabis product for medicinal purposes (*N* = 1059)

Factors	%
Strain type	
- Indica-dominant	25.1
- Sativa-dominant	20.3
- Indica/Sativa blend	25.8
Cannabinoid profile	
- High THC content	47.0
- Low THC content	7.7
- High CBD content	14.7
- THC-CBD equal mix	18.5
Characteristics	
- Taste	33.8
- Smell	19.8
- Look	10.6
Cultivation/Production	
- Organically grown	41.3
- Non-irradiated	15.5
Miscellaneous	
- Strain name (e.g. Kush, Haze)	15.8
- Terpenoid profile	12.7
- Recommended by others	19.0
- Other factors	6.2

Note. Non-irradiated means not sterilized using ionizing radiation with gamma rays

### 6. Prescription medication use

#### Substitution of prescription medication with cannabis

Most participants (77.7%) reported having used prescription medication at some point for the conditions or symptoms they were managing with cannabis, and 40.7% were currently using prescription medication. Among those who had ever used prescription medication, 53.5% reported having substituted cannabis for their medication at least once. These participants reported the types of medications they had substituted by selecting from predefined medication categories developed for this study; participants were not asked to report individual medications. The most commonly substituted medication classes were pain medications (52.7%) and sleep medications (46.7%), followed by antidepressant medications (34.2%), ADHD medications (27.2%), anti-anxiety medications (16.9%), antipsychotic medications (9.7%), arthritis medications (4.4%), anti-seizure medications (1.8%), and other medications (9.3%).

#### Comparison of cannabis and prescription medication use

Participants who had ever used prescription medication were asked to evaluate the impact of cannabis on their medication use and to compare the perceived effectiveness and side effects. Among respondents (*n* = 794), 46.1% reported discontinuing their prescription medication, and 26.3% reported reducing their use since initiating cannabis (Table 8). The majority of participants (75.1%) rated cannabis as slightly or much more effective than their prescribed medication. Similarly, 81.7% indicated that the side effects of their prescription medication were slightly or much worse than those experienced with cannabis.

**Table 8:** Effect of cannabis on prescription medication and compared effectiveness and side effects (*n* = 794)

Items and response options	%
Effect of cannabis use on prescription medication use	
- I stopped using prescription medication	46.1
- I use less prescription medication than before	26.3
- No change	18.1
- I use more prescription medication than before	0.5
- I use another type of medication now	1.9
- Don’t know	7.1
Effectiveness of cannabis compared to prescription medication	
- Cannabis is much more effective	59.4
- Cannabis is slightly more effective	15.7
- No difference	9.3
- Prescription medication is slightly more effective	2.8
- Prescription medication is much more effective	2.3
- Don’t know	10.5
Side effects of cannabis compared to prescription medication	
- Side effects of prescription medication are much worse	71.4
- Side effects of prescription medication are slightly worse	10.3
- No difference	9.7
- Side effects from cannabis are slightly worse	1.9
- Side effects from cannabis are much worse	0.3
- Don’t know	6.4

### 7. Access to prescribed cannabis

#### Accessing prescribed cannabis: steps and barriers

We examined the steps that participants had taken to access prescribed cannabis. Among the total sample, 66.2% reported having discussed their medicinal use of non-prescribed cannabis with a physician, 30.5% had requested a prescription, 9.2% had received a prescription, and 2.3% were currently using prescribed cannabis.

Participants who had never requested prescribed cannabis from a physician (*n* = 736) were asked to indicate their reasons for not doing so. They could select multiple responses from a structured list. The five most frequently reported reasons were: believing their physician would not prescribe cannabis (49.0%), not knowing cannabis could be obtained through a physician (32.5%), concerns about prescribed cannabis being too expensive (24.9%), feeling uncomfortable asking a physician for prescribed cannabis (23.9%), and not wanting to obtain cannabis through a pharmacy (20.9%).

Participants who had requested but not received prescribed cannabis (*n* = 226) were asked to indicate why their physician declined to prescribe it. Multiple responses could be selected from a structured list. The five most commonly reported reasons were: the physician’s lack of knowledge about prescribed cannabis (39.4%), the physician’s assessment that the participant was not eligible for cannabis (31.9%), the physicians’ negative views about cannabis (27.0%), the physician’s scepticism regarding the efficacy of cannabis (26.1%), and the physician’s wish to first explore alternative medications (15.5%).

#### Reasons for using non-prescribed instead of prescribed cannabis

Participants were then asked to indicate up to three reasons for using non-prescribed rather than prescribed cannabis. The most frequently reported reasons were that non-prescribed cannabis was perceived as higher quality (33.1%), less expensive (30.2%), and easier to obtain (29.8%) than prescribed cannabis. Other responses are presented in Table 9.

**Table 9:** Reasons for using non-prescribed rather than prescribed cannabis for medicinal purposes (*n* = 1056)

Reasons	%
Better quality	33.2
Cheaper	30.3
Easier to obtain	29.9
I want to decide how I use cannabis medicinally	25.6
I did not know I could get cannabis from a doctor	21.4
My doctor does not want to prescribe cannabis	18.0
Pharmacies do not have the cannabis products I want	12.8
Pharmacies lack variety in cannabis products	7.2
I do not want my doctor to know I use cannabis medicinally	5.0
Other	9.5

### 8. Experiences with prescribed cannabis

#### Comparison of experiences with non-prescribed and prescribed cannabis

Participants who had used prescribed cannabis in the past (*n* = 72) or were currently using it (*n* = 24) evaluated their experiences using a 5-point Likert scale (see Table 10). Although the sample size was too small for definite conclusions, the findings suggest that this subgroup generally viewed non-prescribed cannabis more favourably than prescribed cannabis. Specifically, 62.9% of respondents agreed or strongly agreed that non-prescribed cannabis was more effective in providing symptom relief than prescribed cannabis, and 60.8% agreed or strongly agreed that it was more pleasant to use, for example in terms of taste or smell. Only a small proportion of participants disagreed with these statements.

**Table 10:** Comparison of non-prescribed and prescribed cannabis (*n* = 96)

Non-prescribed cannabis is…	Strongly agree	Agree	No difference	Disagree	Strongly disagree
More effective (n, %)	46 (47.4%)	15 (15.5%)	30 (30.9%)	3 (3.1%)	3 (3.1%)
More pleasant to use (n, %)	39 (40.2%)	20 (20.6%)	28 (28.9%)	5 (5.2%)	5 (5.2%)

#### Reasons for discontinuing or supplementing prescribed cannabis

Participants who had used prescribed cannabis in the past (*n* = 72) were asked why they stopped using it, while current users of prescribed cannabis (*n* = 24) were asked why they also used non-prescribed cannabis. Responses to these open-ended questions were coded qualitatively, with up to three reasons coded per participant. The two most frequently cited reasons for discontinuing prescribed cannabis were its high cost and/or lack of reimbursement through health insurance (40.5%, *n* = 36), followed by perceptions of lower effectiveness compared to non-prescribed cannabis (27.0%, *n* = 34). Among those currently using prescribed cannabis, the two most common reasons for concurrently using non-prescribed cannabis were its lower cost (29.7%, *n* = 8) and greater perceived effectiveness (25.9%, *n* = 7).

## Discussion

This study is the first to examine patterns of use, motives for use, perceived effectiveness, and access to prescribed cannabis among individuals using non-prescribed cannabis for medicinal purposes in the Netherlands. The findings provide valuable insights into the characteristics, behaviours, and preferences of this population, as well as the barriers they face in accessing prescribed cannabis. These insights can help inform more equitable and patient-centred medical cannabis programs and related policies.

### Cannabis sources and patterns of use

Coffeeshops were the most common source of non-prescribed cannabis, with herbal cannabis as the preferred form. This reflects the distinctive Dutch regulatory context, which tolerates the sale of herbal and resin cannabis in licensed coffeeshops but prohibits extraction-based products. The widespread availability of coffeeshops likely explains their role as the primary access point for medicinal users outside the formal prescription system. However, one in four participants also reported home cultivation. This may reflect both financial pressures and distrust of coffeeshops, where products are not quality controlled and originate from the illicit supply chain. These concerns were evident in our sample, as nearly half of participants reported experiencing a financial burden and more than 40% expressed concerns about contaminants. Similar motivations were identified in international research, where small-scale cultivation of cannabis for medicinal use was often driven by its lower cost and distrust in products from the black market [19]. 

Most participants in our study reported obtaining cannabis from multiple sources and in multiple forms. Some may supplement cannabis purchased from coffeeshops with home cultivation to reduce costs, or they might be able to obtain different product types from different sources. The use of different product types suggests that individuals tailor their use to specific therapeutic needs: inhaled forms are often used for rapid symptom relief, whereas ingested forms are preferred for longer-lasting effects. This adaptive approach underscores the importance of product variety in meeting different treatment goals and patient preferences.

### Routes of administration and harm reduction

Despite international trends toward less harmful consumption methods [12, 20, 21], smoking cannabis mixed with tobacco remains the dominant practice in the Netherlands among both medicinal and recreational users [22]. Participants in this study were more likely than recreational users to adopt less harmful routes of administration [22], which may reflect greater health-consciousness among medicinal users. Harm reduction is especially relevant given the typically near-daily and long-term nature of medicinal cannabis use. Notably, although vaporizing cannabis flower appears more common in the Netherlands than in other European countries [23], it remains one of the least used routes of administration in the Netherlands. This limited uptake may be due to the high cost of vaporizers and the cultural norm of smoking cannabis in a joint. In contrast, sublingual cannabis oil emerged as the second most common mode of consumption among Dutch medicinal users, which may be partly the result of efforts by patient advocacy organizations to provide courses and resources on home preparation of cannabis oil.

Vaporization and oral administration, while not risk-free, are generally considered less harmful than smoking as they avoid combustion-related toxins and nicotine exposure [24]. Nevertheless, both methods have potential drawbacks. In jurisdictions where cannabis concentrates are available, vaporizing these products can produce markedly stronger subjective effects and higher blood THC levels. Oral consumption may result in unintentional overuse, as the delayed onset of effects can prompt users to ingest more than intended. In our study sample, preferences for specific routes of administration were shaped by factors such as convenience, health impact, and the desired pharmacokinetic effects. These findings can help inform harm reduction strategies. For instance, vaporization of plant material may be recommended as a less harmful alternative to smoking for patients seeking rapid symptom relief.

In the Netherlands, medical cannabis is primarily prescribed either as herbal cannabis or sublingual oil, with roughly half of patients using each form [25]. In contrast, countries such as Australia [26], Canada [27], and the United States[28] offer a wider range of prescribed products, including extracts, therapeutic vapes, and oral preparations such as oils, capsules, and edibles. Expanding the range of available formulations in the Netherlands could better accommodate divers therapeutic needs, align treatment options with patient preferences, and encourage less harmful routes of administration. In addition, no data is currently available on the annual quantities of each medical cannabis strain consumed in the Netherlands. Systematically recording this information could provide insight into prescribing trends and inform clinical guidance.

### Limited knowledge of cannabis content

The findings of this study highlight significant concerns regarding the use of unregulated cannabis products for medicinal purposes. More than half of participants reported either not knowing or merely estimating the THC and CBD content of the cannabis they used. Interestingly, about one-fifth of the provided estimates were implausible, revealing considerable gaps in users’ understanding of cannabinoid composition. While some Dutch coffeeshops provide information on the cannabinoid content, many do not. Even when such information is available, research has demonstrated significant discrepancies between labelled and actual THC levels [5]. The lack of knowledge among users is therefore largely due to the absence of standardized testing and labelling in the non-prescribed cannabis market. This uncertainty undermines patients’ ability to self-titrate effectively and increases the risk of over- or under-dosing, a concern that is particularly salient for those managing chronic or complex health conditions.

Participants also expressed concerns about potential contaminants in non-prescribed cannabis. Prescribed cannabis would mitigate this risk, as it is subject to rigorous quality control and ensures consistent content across batches. However, as not all individuals will qualify for prescribed cannabis, it is equally important to enhance the safety and transparency of the broader cannabis market. In that regard, the Dutch Cannabis Experiment – which allows for the controlled cultivation and regulated sale of cannabis – represents a promising step to address these quality and safety concerns [2]. 

### Medical conditions and symptoms managed with cannabis

The majority of participants in our study reported using non-prescribed cannabis to manage physician-diagnosed medical conditions rather than non-specific symptoms, indicating that cannabis is often used for recognized health issues. A wide range of conditions were reported, with chronic pain and mental health conditions among the most common. This pattern is consistent with international research showing diverse motives for medicinal use of cannabis [12–15, 29]. A recent study from the Netherlands, however, found that most patients with prescribed cannabis received it for chronic pain, while few were prescribed it for mental health and other conditions [11]. This points to a misalignment between prescription practices and the therapeutic needs reported by patients.

This mismatch in needs and prescribing practices becomes even more complex when considering the high prevalence of comorbidity among medicinal cannabis users. In our study, approximately three in four participants reported using cannabis for more than one condition. This finding aligns with earlier research demonstrating that patients often seek relief from multiple symptoms or health conditions at the same time [12, 23]. Epidemiological data further supports this pattern, showing that conditions such as chronic pain, depression, anxiety, and sleep disturbances commonly co-occur [30–32]. The high degree of symptom comorbidity contributes to greater care complexity and has important implications for clinical practice. For instance, while physicians may be willing to prescribe cannabis for chronic pain, they often remain reluctant to do so for mental health conditions. This raises questions about how clinicians navigate cannabis prescribing decisions when patients present with multiple indications, underscoring the need for integrated care approaches and clear clinical guidelines for patients presenting with comorbidity.

Although evidence for the effectiveness of cannabis in managing physical health conditions is growing, data on its efficacy for mental health conditions remains limited [33–35]. Patient-reported outcomes from this study and others [36–38] suggest that people with anxiety, depression, PTSD, or other conditions can perceive cannabis as beneficial for symptom relief and overall wellbeing. Given the widespread use of cannabis for mental health concerns globally, further research is urgently needed to elucidate its therapeutic mechanism, rule out potential placebo effects, and assess potential long-term risks [39]. 

### Self-reported effectiveness and other benefits

Participants overwhelmingly reported that cannabis use was effective in improving their symptoms and quality of life. These findings are consistent with other research on patients using prescribed [18, 40, 41], as well as non-prescribed cannabis [12, 42]. Our study also sheds light on the broader positive effects of cannabis on wellbeing and daily functioning, suggesting that the therapeutic value of cannabis extends beyond symptom relief. This reinforces the need to adopt a holistic framework when considering the therapeutic use of cannabis [43]. 

Randomized controlled trials (RCTs) on medical cannabis remain scarce due to a number of factors, including legal and regulatory constraints on cannabis research [44, 45], as well as economic and technical challenges [46]. In the absence of robust RCT-evidence, patient-reported outcomes and other real-world observational data provide an important source of information to guide clinical practice and inform policy decisions [47, 48]. However, findings regarding the self-reported effectiveness of cannabis should still be interpreted with care, as individuals who experience no benefits or adverse effects are less likely to continue using cannabis and are consequently underrepresented in such research. Around 1% of participants in our study reported that cannabis worsened their symptoms and quality of life. Further research is needed to understand the experiences of this subgroup and why they continue to use cannabis despite adverse effects.

### Blurred line between medicinal and recreational use

Our findings highlights the often-blurred line between recreational and medicinal cannabis use. Consistent with previous research [14, 18, 23], a substantial proportion of participants reported using cannabis for both purposes. While legal frameworks often draw a clear line between medicinal and recreational use, research findings challenge this binary perception, suggesting that it does not fully capture user experiences. A key factor contributing to this overlap is the inherently relaxing effect of cannabis, which was also commonly reported by participants. Individuals with conditions such as chronic pain, anxiety, or ADHD may particularly value these relaxing effects. Even if symptoms persist, they may be perceived less or be considered less relevant, helping individuals to function better in daily life and enhance their subjective wellbeing [14]. 

Most participants reported using cannabis recreationally before initiating medicinal use. This suggests that many first experience the therapeutic effects through recreational use. Previous research indicates that individuals who transition from recreational to medicinal use are more likely to use cannabis for mental health conditions and engage in harmful consumption patterns, such as smoking and problematic use [49]. These patterns were also evident in our sample, where many participants reported smoking cannabis and using it for mental health issues. Individuals who first experience the therapeutic effects through recreational use may benefit from additional guidance from healthcare professionals and tailored harm reduction interventions.

### Financial burden

About half of participants reported that their medicinal cannabis use imposed a financial burden. Among those reporting no financial burden, about one-quarter obtained cannabis free of charge. Individuals who did not pay for cannabis were significantly more likely to grow it themselves or make their own oil compared with those who purchased it, suggesting that home cultivation and production may be strategies to reduce costs. Participants who purchased non-prescribed medicinal cannabis reported spending around €100 per month, representing a substantial recurring expense, particularly for those with low disposable income. In the Netherlands, prescribed cannabis is not reimbursed by health insurance providers [10], posing a barrier to equitable access to medical cannabis. This issue is particularly salient given that nearly half of the study sample reported being unable to work. Introducing insurance coverage for prescribed cannabis could mitigate the financial burden and improve access for vulnerable groups [50]. Health insurance models from countries such Germany and the Czech Republic, where coverage is provided for certain medical conditions, may serve as policy guidance [51, 52]. 

### Prescription medication use

Our findings indicate that a substantial proportion of participants considered cannabis a viable alternative to prescription medications. In line with previous studies [17, 18], many reported that cannabis helped reduce or discontinue conventional medications, was often perceived as more effective, and was associated with fewer side effects. These results underscore the need for rigorous economic evaluations to quantify potential healthcare cost savings from reduced prescription drug use. Emerging cost-effectiveness analyses of medical cannabis could inform policy decisions and incentivize health insurances to expand coverage for medical cannabis, thereby lowering financial barriers [53–55]. Substituting cannabis for medications such as opioids or benzodiazepines may also reduce risks of dependence, overdose, and other adverse effects associated with these drug classes. Recent evidence suggests that medical cannabis may offer benefits comparable to opioids for chronic non-cancer pain, while lowering overdose risk and without substantially increasing costs [56]. Further research is needed to evaluate the clinical implications of cannabis substitution in real-world settings.

### Access to and experiences with prescribed cannabis

Our data provides initial insights into barriers to accessing prescribed cannabis in the Netherlands. Approximately two-thirds of participants reported discussing their medicinal use of non-prescribed cannabis with a physician, consistent with previous research [15, 57, 58]. However, one-third had not disclosed their use, and more than one-fifth of the total sample reported being denied a prescription when requested. Reported barriers included physicians’ perceived lack of knowledge about medical cannabis and participants’ concerns about stigma. Similar barriers have been documented in other countries [59–62], underscoring the need for enhanced physician education on the therapeutic use of cannabis. Further qualitative research is warranted to explore these barriers in greater depth from the perspectives of both patients and physicians.

Interestingly, participants who had obtained prescribed cannabis generally reported more favourable experiences with non-prescribed than prescribed cannabis. While this finding needs to be interpreted with caution given the small and potentially biased subsample, it points to possible shortcomings in the current prescribed cannabis system. Across the sample as a whole, participants cited several reasons for using non-prescribed rather than prescribed cannabis, including perceptions of better quality, lower costs, and greater ease of access. Furthermore, they expressed preferences for cannabis products with higher THC content, organically cultivated cannabis, and varieties with pleasant flavour. These patient preferences invite reflection on how cannabis as a medicine differs from conventional pharmaceutical drugs, where sensory factors such as flavour are typically less relevant. Some participants also indicated a preference for non-irradiated products, citing beliefs that irradiation diminishes the therapeutic properties of cannabis or alters its terpene content. This perceptions further supports the finding that individuals choose non-prescribed cannabis because they believe it is of better quality. Taken together, the findings suggest that prescribed cannabis in the Netherlands does not fully meet patient expectations or therapeutic needs, highlighting the importance of a more patient-centred approach.

Currently, only five cannabis strains are available by prescription in the Netherlands, compared with more than 150 strains in countries such as Germany and the United Kingdom [51, 63]. Expanding the range of strains in the Netherlands, with divers cannabinoid and terpenoid profiles, could better accommodate the heterogenous needs of patients and improve clinical outcomes. Unlike conventional pharmaceuticals, cannabis treatment is characterized by substantial variability in product composition, as well as in patient characteristics and preferences, all of which can influence both treatment efficacy and uptake.

Despite differences in national cannabis policies and the length of time that medical cannabis has been available, the use patterns, motives for use, and barriers to accessing prescribed cannabis observed in this study are broadly consistent with findings from other countries [12–15, 17, 18]. These similarities highlight opportunities for cross-national learning that can inform policy and clinical practice, with the aim of improving access to prescribed cannabis for eligible patients and enhancing patient care.

### Limitations

This study has several limitations that should be considered when interpreting the findings. First, there is potential for selection bias. Individuals who experience limited or negative effects from medicinal cannabis are less likely to participate in research, which could lead to an overrepresentation of positive experiences. The sample is also likely skewed toward individuals with internet access and sufficient time or resources to complete an online survey, which may limit generalizability. Second, the recruitment strategy may have introduced sampling biases. Most recruitment resources were allocated to Facebook advertisements. While this method is cost-effective and efficient for reaching a broad audience [64, 65], Facebook’s algorithmic targeting may have preferentially reached certain demographic groups [66]. Individuals who do not use Facebook were inherently excluded. Consequently, the sample may not fully represent the broader population of medicinal cannabis in the Netherlands. Third, all data was self-reported, introducing the possibility of recall bias, social desirability bias, or confirmation bias. Participants may have overstated the efficacy of cannabis or underreported adverse effects.

Fourth, inaccuracies may have been introduced if participants misunderstood or did not fully adhere to the survey instructions. For example, participants were asked to indicate the conditions they managed with cannabis, but some may have listed all their medical conditions regardless of whether they used cannabis for it. Furthermore, no medical histories were collected, so participants’ reported diagnoses could not be verified. Fifth, although obvious duplicate responses were removed, the possibility of undetected duplicates cannot be ruled out, because IP-addresses or other identifying information were not accessible to the researchers. Sixth, the study excluded individuals using commercially available CBD products, due to the lack of evidence supporting their efficacy and lower CBD concentrations compared with approved CBD medications [67]. Given that approximately half of self-identified medicinal cannabis users in the Netherlands report using CBD oils [3], future research should investigate the efficacy and patterns of use of these widely available commercial CBD products.

## Conclusions

Our findings indicate that people using non-prescribed cannabis for medicinal purposes represent a heterogenous population, often using multiple cannabis products from diverse sources to manage a range of health conditions simultaneously. This population faces considerable health risks: most continue to smoke cannabis with tobacco, have limited knowledge of THC and CBD content, and experience substantial barriers to accessing prescribed cannabis. Expanding access to prescribed cannabis could support harm reduction by reducing exposure to unregulated or potentially contaminated products, promoting less harmful consumption methods, and enabling clinical monitoring of treatment efficacy and adverse effects. To facilitate the transition from the unregulated market to the regulated healthcare system, prescribed cannabis programs and policies should adopt a patient-centred approach that integrates the latest scientific evidence while accounting for the diverse therapeutic needs and preferences of users. The findings of this study offer valuable insights into patterns of use and user preferences, which can help guide the development of more effective and equitable medical cannabis policies and practices.

## Supplementary Information

Supplementary Material 1

## Data Availability

The datasets generated and analysed during the current study are not publicly available to protect individual privacy but are available from the corresponding author on reasonable request. The questionnaire is provided in the Supplementary Material.

## Abbreviations

CBD: cannabidiol
ROA: routes of administration
THC: tetrahydrocannabinol
QOL: quality of life
RCT: randomized controlled trial

## Footnotes

1 A ‘coffeeshop’ is an establishment where the sale of cannabis products (herbal cannabis and cannabis resin) in small quantities for personal consumption is tolerated by the Dutch authorities. This means that although the sale is a criminal offence, the Public Prosecution Service does not prosecute coffeeshops for this [68].

2 Responses in the open-text field ‘other sources’ were coded and a new category was generated if 35 or more participants gave similar answers. Two new categories were added: ‘non-cannabis related websites’ and ‘personal network’.

3 The European Union Drugs Agency (EUDA) defines ‘daily or near-daily use’ as 20 days or more in a month [69].

4 At the time of this study, cannabis in the Netherlands was available only through the illicit market. Although the sale of cannabis is illegal, it is tolerated in licensed coffeeshops by the Dutch government. In 2025, legally produced cannabis became available in ten municipalities as part of the Cannabis Closed Supply Chain Experiment, which requires products to display cannabinoid concentrations on their labels. However, this experiment began about two years after data collection for this study ended.

5 To ensure national comparability, we used the same list of medical conditions as a recent study on prescribed medical cannabis patients in the Netherlands [11].

6 Responses in the ‘Other’ category were reviewed and any conditions that clearly matched existing categories were recoded accordingly.
