# Supplementary material for: Medicinal use of non-prescribed cannabis: a cross-sectional survey on patterns of use, motives for use, and treatment access in the Netherlands
Source: J Cannabis Res. 2025 Dec 2;8:6. doi: 10.1186/s42238-025-00355-y (PMC12777223; doi:10.1186/s42238-025-00355-y)
Supplement: Supplementary file 1 — Supplementary Material 1 [file 42238_2025_355_MOESM1_ESM.docx]

**Supplementary Material** for ‘Medicinal use of non-prescribed cannabis: a cross-sectional survey on patterns of use, motives for use, and treatment access in the Netherlands’

- Supplementary Material S1. Original Dutch version of the questionnaire
- Supplementary Material S2. English translation of the Dutch questionnaire
  - Please note: This translation was produced by the authors and has not undergone professional validation.

**Supplementary Material S1. Original Dutch version of the questionnaire**

**Welkom bij de MEDUSA vragenlijst**

Gebruik je wiet, hasj, of wietolie om lichamelijke of psychische klachten te verminderen?

En haal je deze cannabisproducten bij de coffeeshop, maak of kweek je ze zelf, of krijg je ze van familie/vrienden?

Dan kun je deze vragenlijst invullen.

Door jouw ervaringen te delen komt er meer kennis beschikbaar over hoe cannabis als medicijn wordt gebruikt. Zo kunnen we proberen de toegang tot medicinale cannabis en het aanbod ervan te verbeteren, zodat het beter aansluit bij de behoeften van patiënten.

Het invullen van de vragenlijst duurt 10 tot 15 minuten. Je antwoorden zijn anoniem.

Als je meedoet maak je kans op één van de 10 geldprijzen van 200 euro.

Uitgebreidere informatie over het onderzoek vind je **hier** terug. Lees deze pagina goed door. Op die pagina vind je ook informatie over de vervolgonderzoeken waaraan je later kunt meedoen.

Voor vragen over dit onderzoek kun je mailen met Lisa Strada (lstrada@trimbos.nl) of Pieter Oomen (poomen@trimbos.nl) van het Trimbos-instituut.

**Toestemming**

Ik ben goed geïnformeerd over het onderzoek én de wijze waarop mijn persoonsgegevens worden verwerkt.

Vragen die ik nog had over het onderzoek en over de verwerking van persoonsgegevens heb ik kunnen stellen, en zijn naar tevredenheid beantwoord.

Ik weet dat meedoen vrijwillig is. Ik weet ook dat ik op ieder moment kan stoppen met deelname aan het onderzoek en dat ik de toestemming voor de verwerking van persoonsgegevens kan intrekken.

Ik stem in met deelname aan het onderzoek en stem in met de verwerking van mijn persoonsgegevens, waaronder gezondheidsgegevens, voor dit onderzoek zoals beschreven in de **informatiebrief** en **privacyverklaring**.

- Ja ik stem in.

**Heb je dit kalenderjaar eerder meegedaan met een onderzoek van het Trimbos-instituut en daarvoor een vergoeding ontvangen?**

- Ja
- Nee

**Eerst een paar korte vragen…**

**Waar heb je deze vragenlijst gevonden?**

- Facebook
- Andere sociale media (bijvoorbeeld Twitter, Instagram)
- Coffeeshop
- Website over cannabis
- Cannabis Social Club (een non-profit stichting waar cannabis wordt gekweekt en gedeeld)
- Anders, namelijk: ______

**Gebruik je cannabisproducten voor lichamelijke of psychische klachten?**

- Ja
- Nee

**Hoe kom je aan de cannabisproducten die je als medicijn gebruikt?**

- Van de dokter
- Niet van de dokter
- Zowel van de dokter als van ergens anders

**Ben je 18 jaar of ouder?**

- Ja
- Nee

**Woon je in Nederland?**

- Ja
- Nee

**Gebruik je *alleen* CBD-producten (zonder THC) die je koopt bij een drogisterij of internet?**

*Als je alleen zulke CBD-producten gebruikt, kun je helaas je niet meedoen aan de vragenlijst.*

- Ik gebruik *alleen* CBD-producten van een drogisterij of internet.
- Nee, ik gebruik (ook) andere cannabisproducten als medicijn, zoals wiet, hasj, en wietolie.

**Algemene informatie over jou**

**Geslacht:**

- Man
- Vrouw
- Anders
- Zeg ik liever niet

**Leeftijd:**

Ik ben [getal] jaar oud.

**Hoogst behaalde opleiding:**

- Basisonderwijs
- Vmbo, mbo1, praktijkonderwijs, onderbouw havo/vwo
- Havo, vwo, mbo
- Hbo, wo bachelor
- Wo master, doctor
- Niet van toepassing/weet ik niet

**Werk op dit moment:**

- Fulltime
- Parttime
- Werkloos
- Met pensioen
- Arbeidsongeschikt/ziek/invalide
- Student

De volgende vragen gaan over de cannabisproducten die je als medicijn gebruikt en die je **niet** van de dokter of de apotheek krijgt.

**Waar haal je de cannabisproducten die je als medicijn gebruikt?**

*Meerdere antwoorden mogelijk.*

- Coffeeshop
- Cannabis Social club (een non-profit stichting waar cannabis wordt gekweekt en gedeeld)
- Ik kweek de cannabis zelf
- Ik maak de wietolie zelf
- Ik krijg het van een vriend of familielid
- Online winkel
- Dealer
- Anders, namelijk: _____

*Toon deze vraag als meer dan een bolletje is aangekruist:*

Waar haal je het vaakst je cannabisproducten vandaan? < *Uitklap keuzelijst: bovenstaande opties tonen* >

**Welke cannabisproducten gebruik je als medicijn?**

*Meerdere antwoorden mogelijk.*

- Wiet
- Hasj
- Cannabisolie die ik via de mond neem of onder mijn tong leg
- Cannabisolie om in te ademen of te verdampen
- Cannabisproducten om te smeren, zoals crème/lotion/zalf
- Anders, namelijk: ____

*Toon deze vraag als meer dan een bolletje is aangekruist:*

Welk cannabisproduct gebruik je het meest? < *Uitklap keuzelijst: bovenstaande opties tonen* >

**Hoeveel THC en CBD zit in het cannabisproduct dat je gebruikt?**

*Geef antwoord voor het cannabisproduct dat je het meest gebruikt.*

THC:

- Veel
- Gemiddeld
- Weinig
- Weet ik niet

CBD:

- Veel
- Gemiddeld
- Weinig
- Weet ik niet

**Hoeveel procent THC en CBD zit er in je cannabisproduct? Als je het niet weet, ga dan naar de volgende vraag.**

THC: ____ %

CBD: ____ %

**Hoe weet je hoeveel THC en CBD er in je cannabisproduct zit?**

- Het personeel in de coffeeshop vertelde het me
- Het stond op het etiket van het cannabisproduct
- Degene die mij het cannabisproduct heeft gegeven of verkocht vertelde het me
- Het is mijn eigen schatting
- Ik weet niet hoeveel THC en CBD er in zit
- Anders, namelijk: ____

**Hoe vaak gebruik je cannabis als medicijn?**

Gemiddeld op [getal] dagen per maand. (Een maand heeft 30 dagen.)

**Op welke manier gebruik je cannabis als medicijn?**

*Meerdere antwoorden mogelijk.*

- Ik rook cannabis met tabak (joint)
- Ik rook cannabis puur (zonder tabak)
- Ik verdamp de cannabis (de bloemen/ toppen)
- Ik verdamp cannabisolie/extract
- Ik doe cannabisolie onder mijn tong
- Ik eet of drink het
- Ik gebruik cannabis crème/lotion/zalf
- Anders, namelijk: ____

*Toon deze vraag als meer dan een bolletje is aangekruist:*

Op welke manier gebruik je cannabis het vaakst? < *Uitklap keuzelijst: bovenstaande opties tonen* >

**Waarom gebruik je de cannabis op die manier?**

*Kies maximaal drie redenen.*

- Het begint snel te werken
- Het werkt lang
- Ik kan makkelijk bepalen hoeveel ik neem
- Gemakkelijk te gebruiken
- Prettig om in te ademen/ te inhaleren
- Minder of niet schadelijk voor de longen
- Goede smaak
- Minder bijwerkingen
- Gemakkelijk te verbergen (bijvoorbeeld buiten of voor de politie)
- Uit gewoonte
- Anders, namelijk: _____

**Hoe lang gebruik je cannabis al als medicijn?**

*Als je het niet meer weet, ga dan naar de volgende vraag.*

____ jaren

**Hoe vaak gebruik je cannabis alleen voor plezier en niet voor je klachten?**

- Nooit
- Heel soms
- Soms
- Vaak

**Hoe vaak gebruikte je cannabis voor je plezier voordat je het als medicijn ging gebruiken?**

- Nooit
- Heel soms
- Soms
- Vaak

**Redenen om cannabis als medicijn te gebruiken**

**Voor welke lichamelijke ziektes of psychische aandoeningen gebruik je cannabis? Hiermee bedoelen we ziektes of aandoeningen die door een dokter zijn vastgesteld.**

*Als je geen diagnose van een dokter hebt, maar cannabis wel gebruikt om bepaalde klachten te verminderen, ga dan naar de volgende vraag.*

- Chronische pijn
- Fibromyalgie
- Kanker
- Multiple sclerose (MS)
- Ziekte van Crohn
- Migraine
- Gilles de la Tourette
- Glaucoom
- Epilepsie
- Ziekte van Parkinson
- Hepatitis C
- HIV/AIDS
- Slaapstoornis
- ADHD/ADD
- Depressie
- Angststoornis
- Posttraumatische stressstoornis (PTSS)
- Autismespectrumstoornis (ASS)
- Anders, namelijk: __________

**Voor welke lichamelijke of psychische klachten gebruik je cannabis?**

- Langdurige (chronische) pijn
- Kortdurende (acute) pijn
- Zenuwpijn
- Misselijkheid en/of braken
- Verbeteren van de eetlust
- Verminderen van bijwerkingen door medicijnen
- Epileptische aanvallen
- Spasticiteit
- Spierpijn en krampen
- Tics
- Tumorgroei tegengaan
- Verhoogde oogdruk
- Premenstrueel syndroom (PMS)
- Slaapproblemen
- Depressieve klachten
- Angsten
- Stress/nervositeit
- Anders, namelijk: _______

**Jouw ervaring met cannabis**

**Hoeveel verbetert cannabis je klachten?**

*Geef een cijfer: 1 = geen verbetering; 10 = volledige verbetering.*

*Als je klachten door cannabis erger worden, kies dan de optie 'Cannabis verergert mijn klachten.'*

[Plaats hier een 10-puntschaal: 1-10]

Optie: Cannabis verergert mijn klachten.

**Zorgt het gebruik van cannabis als medicijn ervoor dat je je op andere manieren ook beter voelt?**

- Ja
- Nee

**Zo ja, kruis alles aan wat van toepassing is:**

- Minder angst
- Minder depressief
- Ik kan beter met mensen omgaan
- Ik kan me beter bewegen en actief zijn
- Ik kan beter werken
- Ik heb meer zin om dingen te doen
- Ik kan me beter ontspannen
- Ik kan me beter concentreren
- Ik slaap beter
- Ik eet beter
- Anders, namelijk: _________

**Hoeveel verbetert het gebruik van cannabis als medicijn je kwaliteit van leven?**

*Geef een cijfer: 1 = geen verbetering; 10 = volledige verbetering.*

*Als je kwaliteit van leven door cannabis slechter is geworden, kies dan de optie ‘Cannabis verslechtert mijn kwaliteit van leven.’*

[Plaats hier een 10-puntschaal: 1-10]

Optie: Cannabis verslechtert mijn kwaliteit van leven.

**Heb je ooit medicijnen van de dokter gekregen voor je ziektes/klachten?**

- Ja
- Nee

**Krijg je op dit moment medicijnen van de dokter voor je ziektes/klachten?**

- Ja
- Nee

*Als JA op ‘ooit medicijnen gebruikt’, toon dan de volgende vragen. Als NEE, stuur de deelnemer door naar het volgende onderdeel: ‘ervaring met de zorg’.*

**Heb je ooit cannabis gebruikt in plaats van een medicijn dat je van de dokter hebt gekregen?**

- Ja
- Nee

**Zo ja, welke medicijnen van de dokter heb je vervangen door cannabis?**

- Medicijnen tegen pijn
- Medicijnen tegen depressie (antidepressiva)
- Medicijnen tegen angst
- Medicijnen tegen psychoses (antipsychotica)
- Medicijnen tegen ADHD
- Medicijnen tegen epilepsie
- Medicijnen tegen artritis
- Medicijnen om te slapen
- Anders, namelijk: ____

**Hoe heeft cannabis het gebruik van de medicijnen van de dokter beïnvloed?**

- Ik ben gestopt met het gebruik van de medicijnen die ik van de dokter kreeg.
- Ik gebruik de medicijnen van de dokter minder dan eerst.
- Er is niks veranderd. Ik gebruik de medicijnen van de dokter op dezelfde manier.
- Ik gebruik nu meer medicijnen van de dokter dan eerst.
- Ik gebruik nu een ander soort medicijn van de dokter.
- Weet ik niet.

**Werkt cannabis beter of slechter dan de medicijnen van de dokter om je klachten te verminderen?**

- Cannabis werkt veel beter dan de medicijnen van de dokter.
- Cannabis werkt iets beter dan de medicijnen van de dokter.
- Cannabis werkt even goed als de medicijnen van de dokter.
- De medicijnen van de dokter werken iets beter dan cannabis.
- De medicijnen van de dokter werken veel beter dan cannabis.
- Weet ik niet.

**Zijn de bijwerkingen van medicijnen van de dokter erger dan de bijwerkingen van cannabis?**

- De bijwerkingen van de medicijnen zijn veel erger dan die van cannabis.
- De bijwerkingen van de medicijnen zijn iets erger dan die van cannabis.
- Er is geen verschil in bijwerkingen.
- De bijwerkingen van cannabis zijn iets erger dan die van de medicijnen.
- De bijwerkingen van cannabis zijn veel erger dan die van de medicijnen.
- Weet ik niet.

**Ervaring met de zorg**

Heb je ooit met een dokter gesproken over dat je cannabis als medicijn gebruikt? Ja/Nee

Heb je ooit aan een dokter gevraagd om je cannabis als medicijn te geven? Ja/Nee

Heb je ooit cannabis als medicijn van de dokter gekregen? Ja/Nee

Gebruik je op dit moment cannabis die je van de dokter hebt gekregen? Ja/Nee

*Als NEE bij vraag 2:*

**Ik heb nog nooit een dokter gevraagd om mij cannabis als medicijn te geven omdat:**

*Meerdere antwoorden mogelijk.*

- Ik wist niet dat ik cannabis als medicijn van de dokter kon krijgen.
- Ik denk niet dat mijn dokter mij cannabis als medicijn zou geven.
- Ik denk dat mijn dokter niet zou weten welke cannabis hij aan mij moet geven.
- Ik vind het niet prettig om mijn dokter om cannabis als medicijn te vragen.
- Ik wil niet dat mijn dokter weet dat ik cannabis als medicijn gebruik.
- Ik wil geen cannabis uit de apotheek.
- Ik heb geen dokter nodig; ik weet welke cannabis het beste voor mij is.
- Cannabis uit de apotheek is te duur.
- Anders, namelijk: ___________

*Als JA bij vraag 2 en NEE bij vraag 3:*

**Mijn dokter heeft me geen cannabis als medicijn gegeven omdat:**

*Meerdere antwoorden mogelijk.*

- Mijn dokter zei dat mijn ziekte/aandoening niet in aanmerking komt voor medicinale cannabis.
- Mijn dokter wist niet genoeg over cannabis als medicijn om het aan mij te geven.
- Mijn dokter denkt dat cannabis iets slechts is.
- Mijn dokter wilde eerst andere medicijnen proberen.
- Mijn dokter denkt dat cannabis als medicijn niet goed werkt.
- Anders, namelijk: _________

*Als JA bij vraag 3 en NEE bij vraag 4:*

**Je hebt laten weten dat je vroeger cannabis van de dokter hebt gekregen, maar het nu niet meer gebruikt.**

Wat voor soort cannabisproduct heb je toen gekregen van de dokter? (Naam, percentage THC en CBD) ______

Waarom ben je gestopt met het gebruiken van cannabis die je van de dokter kreeg? ____________

*Als JA bij vraag 4:*

**Je hebt laten weten dat je op dit moment cannabis van de dokter gebruikt.**

Wat voor soort cannabisproduct krijg je van de dokter? (Naam, percentage THC en CBD) _________

Waarom gebruik je ook andere cannabis, die je niet van de dokter krijgt, als medicijn? ____________

*Toon deze items aan respondenten die de laatste 2 vragen over ‘MC verleden’ of ‘MC huidig’ hebben ingevuld:*

**Hoeveel ben je het eens of oneens met de volgende uitspraken?**

***Cannabis die ik zelf kan halen (zonder doktersrecept) werkt beter tegen mijn klachten dan de cannabis die ik van de dokter kreeg/krijg.***

- Helemaal mee eens
- Mee eens
- Ze werken even goed
- Mee oneens
- Helemaal mee oneens

***Cannabis die ik zelf kan halen is prettiger om te gebruiken dan de cannabis die ik van de dokter kreeg/krijg (de smaak of de geur is bijvoorbeeld prettiger).***

- Helemaal mee eens
- Mee eens
- Ze zijn even prettig om te gebruiken
- Mee oneens
- Helemaal mee oneens

**Wil je nog iets zeggen over je ervaring met cannabis die je van de dokter kreeg/krijgt?** ______

*(Niet-verplicht item)*

*Toon deze items weer aan iedereen.*

**Waarom gebruik je cannabis die je zelf kunt halen (zonder doktersrecept) in plaats van cannabis die je van de dokter kunt krijgen?**

*Kies maximaal 3 redenen.*

- Cannabis die ik zelf kan halen is goedkoper
- Cannabis die ik zelf kan halen is makkelijker te verkrijgen
- Cannabis die ik zelf kan halen is van betere kwaliteit
- Apotheken hebben niet de cannabisproducten die ik wil
- Apotheken hebben niet genoeg verschillende soorten cannabisproducten
- Mijn dokter wil mij geen recept voor medicinale cannabis geven
- Ik wil niet dat mijn dokter weet dat ik cannabis als medicijn gebruik
- Ik wil zelf bepalen hoe ik cannabis als medicijn gebruik
- Ik wist niet dat ik cannabis als medicijn van de dokter kon krijgen
- Anders, namelijk: _____

**Heb je wel eens slechte dingen gehoord over de medicinale cannabis die je van de dokter kunt krijgen?**

Zo ja, schrijf op wat je hebt gehoord: ________

*(Niet-verplicht item)*

**Wat voor soort cannabis gebruik je het liefst?**

**Ik vind de volgende dingen belangrijk als ik de cannabis kies die ik als medicijn gebruik.**

*Kies maximaal vijf.*

- Indica-dominant
- Sativa-dominant
- Indica/Sativa mix
- Veel THC
- Weinig THC
- Veel CBD
- THC:CBD gelijke mix
- Wat voor soort terpenen erin zitten
- Hoe de bloem ruikt
- Hoe de bloem eruit ziet
- Naam (bijv. Kush, Haze)
- Smaak
- Biologisch geteeld (zonder chemische bestrijdingsmiddelen)
- Niet bestraald
- Ik vertrouw op wat anderen mij vertellen (bijvoorbeeld coffeeshoppersoneel, vrienden, internet)
- Anders, namelijk: _____

**Kosten en stress**

**Hoeveel geef je ongeveer per MAAND uit aan de cannabisproducten die je als medicijn gebruikt?**

- _____ Euro’s per maand (vul hier in)
- Ik betaal niet voor mijn cannabis.
- Zeg ik liever niet.

**Heb je geldzorgen omdat je cannabis als medicijn gebruikt?**

- Helemaal niet
- Een beetje
- Enigszins
- Heel erg

**Maak je je wel eens zorgen over je gebruik van cannabis als medicijn als het gaat om de volgende dingen:**

*Beantwoord elk onderdeel*

De illegale status van cannabis Ja/Nee

Stigma (slechte imago) Ja/Nee

Gezondheidsproblemen (bijvoorbeeld met de longen) Ja/Nee

Vervuiling (bijvoorbeeld met pesticiden) Ja/Nee

Onzekerheid of mijn cannabisproduct altijd beschikbaar is Ja/Nee

Kans op verslaving Ja/Nee

**Wil je nog iets zeggen over het gebruik van cannabis als medicijn? Of over deze vragenlijst?** ____

*(Niet-verplicht item)*

Hartelijk dank voor het invullen van deze vragenlijst!

Zo meteen kun je meedoen aan een loterij. Daarmee maak je kans op 200 euro.

Je kunt je eerst nog aanmelden voor vervolgonderzoeken van dit project. Dit is vrijwillig.

Als je mee wilt doen aan één of beide vervolgonderzoeken, dan vragen we om je e-mailadres. In de **privacyverklaring** vind je meer informatie over hoe we met je gegevens omgaan en welke rechten je hebt.

Ken je iemand die cannabis als medicijn gebruikt? Stuur deze link alsjeblieft door: <https://trimbos.nl/medusa/>

Hoe meer mensen hun ervaringen delen hoe beter!

**Vervolgonderzoek 1: Een Interview.**

We willen graag van je weten:

Waarom haal je de cannabis die je als medicijn gebruikt niet van de dokter?

Wat vind je prettig of niet prettig aan de manier waarop je nu aan je cannabis komt?

Het interview duurt 45-60 minuten en gebeurt online. Wie meedoet krijgt een **vergoeding van 40 euro.**

In de informatiebrief en in de privacyverklaring vind je meer informatie over het onderzoek, over hoe we met je gegevens omgaan en welke rechten je hebt.

**Heb je interesse om deel te nemen?**

- Ja, ik wil graag meer informatie krijgen over het onderzoek. Mijn emailadres is: ______
- Nee

**Vervolgonderzoek 2: Je wiet/hasj opsturen.**

We willen weten hoeveel THC en CBD in de wiet/hasj zit die je voor je klachten gebruikt.

Zo weten we welke soort cannabisproducten patiënten het liefst gebruiken.

Om precies te weten hoeveel THC en CBD er in je wiet/hasj zit, hebben wij 1,5 gram van je wiet of hasj nodig.

Je krijg **40 euro als vergoeding** als je meedoet**.**

In de informatiebrief en in de privacyverklaring vind je meer informatie over het onderzoek, over hoe we met je gegevens omgaan en welke rechten je hebt.

**Heb je interesse om deel te nemen?**

- Ja, ik wil graag meer informatie krijgen over het onderzoek. Mijn emailadres is: ______
- Nee

Je kunt nu meedoen aan een loterij om kans te maken op één van de 10 prijzen van 200 euro.

Klik om mee te doen op **DEZE LINK**. Dan kom je op een pagina waar je je e-mailadres kunt invullen.

Als je niet wilt deelnemen aan de loterij klik dan op **Volgende** om de vragenlijst af te ronden.

Vul hier je e-mailadres in om mee te doen aan de loterij.

E-mail: ______

We stellen je binnen 4 maanden per e-mail op de hoogte als je hebt gewonnen.

Hartelijk dank voor je deelname aan dit onderzoek!

**Supplementary Material S2. English translation of the Dutch questionnaire**

**Welcome to the MEDUSA questionnaire**

Do you use weed, hash, or cannabis oil to alleviate physical or psychological symptoms?

And do you get these cannabis products from a coffeeshop, make or grow them yourself, or receive them from family or friends?

If so, you can take part in this survey.

By sharing your experiences, you provide valuable insight into how cannabis is used as medicine. This information can help improve access to medical cannabis and ensure that cannabis products better meet patients’ needs.

The survey takes about 10 to 15 minutes to complete. Your answers will remain anonymous.

By participating, you also have a chance to win one of ten cash prizes of 200 Euro.

You can find more detailed information about the study on **this** page. Please read it carefully. On that page you will also find information about follow-up studies that you can join later.

If you have any questions about this research, you can contact Lisa Strada (lstrada@trimbos.nl) or Pieter Oomen (poomen@trimbos.nl) at the Trimbos Institute.

**Consent**

I have been well informed about the study and about how my personal data will be processed.

Any questions I had about the study or the use of my personal data have been answered to my satisfaction.

I understand that participation is voluntary. I also know that I can stop taking part at any time and withdraw my consent for the use of my personal data.

I agree to take part in this study and to the processing of my personal data, including health data, for this study as described in the **information letter** and **privacy statement**.

- Yes, I give consent.

**Have you already taken part in a Trimbos Institute study this calendar year and received compensation for it?**

- Yes
- No

**First a few short questions…**

**Where did you find this survey?**

- Facebook
- Other social media (for example Twitter, Instagram)
- Coffeeshop
- Website about cannabis
- Cannabis Social Club (a non-profit organization where cannabis is grown and shared)
- Other, namely: ______

**Do you use cannabis products for physical or psychological symptoms?**

- Yes
- No

**How do you obtain the cannabis products you use as medicine?**

- With a medical prescription
- Without a medical prescription
- Both with and without a medical prescription

**Are you 18 years or older?**

- Yes
- No

**Are you resident in the Netherlands?**

- Yes
- No

**Do you *only* use non-prescribed CBD products (without THC) that you can buy from a drug store or online?**

*If you only use these CBD products, unfortunately you cannot take part in this survey.*

- I only use non-prescribed CBD products from a drug store or online shop.
- No, I (also) use other cannabis products as medicine, such as weed, hashish, or cannabis oil.

**General information about you**

**Gender:**

- Male
- Female
- Other
- Prefer not to say

**Age:**

I am [number] years old.

**Highest level of education completed:**

- Primary school
- Lower secondary education/ pre-vocational secondary education
- Higher secondary education/ vocational secondary training
- Bachelor’s degree (or equivalent)
- Master’s degree or Doctorate
- Not applicable/ I don’t know

**Current work situation:**

- Full-time
- Part-time
- Unemployed
- Retired
- Unable to work due to illness/disability
- Student

The next questions are about the cannabis products you use for medicinal purposes and for which you do **not** have a medical prescription.

**Where do you obtain the cannabis products you use for medicinal purposes?**

*Choose all that apply.*

- Coffeeshop
- Cannabis Social club (a non-profit foundation where cannabis is grown and shared)
- I grow cannabis myself
- I make cannabis oil myself
- I get it from a friend or family member
- Online shop
- Dealer
- Other: _____

*Show this question if more than 1 response was selected:*

What is your main source of cannabis? *< Drop down list with the options above >*

**What cannabis products do you use for medicinal purposes?**

*Choose all that apply.*

- Herbal cannabis
- Hashish
- Cannabis oil for oral or sublingual use
- Cannabis oil to inhale or vaporize
- Cannabis products for the skin, such as cream/lotion/ointment
- Other: ____

*Show this question if more than 1 response was selected:*

Which cannabis product do you use the most? *< Drop down list with the options above >*

**How much THC and CBD is in the cannabis product that you use for medicinal purposes?**

*Please provide an answer for the cannabis product that you use the most.*

THC-level:

- High
- Medium
- Low
- I don’t know

CBD-level:

- High
- Medium
- Low
- I don’t know

**What percentage THC and CBD is in your cannabis product? If you don’t know, skip this question.**

THC: ____ %

CBD: ____ %

**How do you know the amount of THC and CBD in your cannabis product?**

- The coffeeshop staff told me
- It was on the label of the cannabis product
- The person who gave or sold the cannabis product to me told me
- It is my own estimate
- I don’t know the THC and CBD content
- Other: _____

**How often do you use cannabis for medicinal purposes?**

On average, on [number] days per month. (A month has 30 days.)

**How do you consume your cannabis as medicine?**

*Choose all that apply.*

- I smoke cannabis with tobacco (joint)
- I smoke cannabis pure (without tobacco)
- I vaporize the cannabis flower/buds
- I vaporize cannabis oil/extract
- I place cannabis oil under my tongue
- I eat or drink it
- I use cannabis cream/lotion/ointment
- Other: ____

*Show this question if more than 1 response was selected:*

Which method do you use most often? *< Drop down list with the options above >*

**Why do you choose this method of consumption?**

*Choose up to three reasons.*

- Quick onset of effects
- Long-lasting effects
- Easy to dose
- Easy to use
- Pleasant to inhale
- Less or not harmful for the lungs
- Good taste
- Fewer side effects
- Easy to hide (for example in public or from the police)
- Out of habit
- Other: _____

**How long have you been using cannabis for medicinal purposes?**

*If you don’t remember, skip this question.*

______ years

**How often do you use cannabis just recreationally?**

- Never
- Rarely
- Sometimes
- Often

**How often did you use cannabis recreationally before you started using it medicinally?**

- Never
- Rarely
- Sometimes
- Often

**Reasons for using cannabis as medicine**

**For which physical or mental health conditions do you use cannabis? By this we mean illnesses or conditions that have been diagnosed by a doctor.**

*If you don't have a medical diagnosis from a doctor, but you do use cannabis to relieve certain symptoms, please go to the next question.*

- Chronic pain
- Fibromyalgia
- Cancer
- Multiple sclerosis (MS)
- Chron’s disease
- Migraine
- Gilles de la Tourette
- Glaucoma
- Epilepsy
- Parkinson’s disease
- Hepatitis C
- HIV/AIDS
- Sleep disorder
- ADHD/ADD
- Clinical depression
- Anxiety disorder
- Post-traumatic stress disorder (PTSD)
- Autism Spectrum Disorder (ASD)
- Other: _______

**For which physical or mental health symptoms do you use cannabis?**

- Long-term (chronic) pain
- Short-term (acute) pain
- Nerve pain
- Nausea and/or vomiting
- Improving appetite
- Reducing side effects from other medication
- Seizures
- Spasticity
- Muscle aches and cramps
- Tics
- Preventing tumour growth
- Increased eye pressure
- Premenstrual syndrome (PMS)
- Sleep problems
- Depressive symptoms
- Anxiety
- Stress/nervousness
- Other: _____

**Your experience with cannabis**

**How much does cannabis improve your symptoms?**

*Rate it on a scale from 1 (no improvement) to 10 (complete improvement).*

*If cannabis makes your symptoms worse, choose the option 'Cannabis makes my symptoms worse.'*

[Insert a 10-point scale: 1-10]

Option: Cannabis makes my symptoms worse.

**Does using cannabis for medicinal purposes also have other positive effects on your wellbeing?**

- Yes
- No

**If Yes, choose all that apply:**

- Less anxiety
- Less depression
- Improved quality of my social interactions
- Improved ability to move and be physically active
- Improved ability to work
- More motivated to do things
- More relaxed
- More focused
- Improved sleep
- Improved appetite
- Other: ________

**How much does using cannabis for medicinal purposes improve your quality of life?**

*Rate it on a scale from 1 (no improvement) to 10 (complete improvement).*

*If your quality of life has become worse because of cannabis, choose the option 'Cannabis makes my quality of life worse.’*

[Insert a 10-point scale: 1-10]

Option: Cannabis makes my quality of life worse.

**Have you ever used prescription medication for your symptoms?**

- Yes
- No

**Are you currently using prescription medication for your symptoms?**

- Yes
- No

*If YES to ‘ever used prescription medication’, show the following questions. If NO, redirect the respondent to the next section on ‘experience with healthcare’.*

**Have you ever used cannabis instead of a medication prescribed by your doctor?**

- Yes
- No

**If yes, which prescription medications did you replace with cannabis?**

- Pain medication
- Antidepressants
- Anti-anxiety medication
- Antipsychotics
- ADHD medication
- Epilepsy medication
- Arthritis medication
- Sleep medication
- Other: ____

**How has cannabis affected your use of prescription medication?**

- I stopped using the prescription medication.
- I use the prescription medication less than before.
- Nothing has changed. I use the prescription medication the same as before.
- I use more prescription medication than before.
- I now use a different type of prescription medication.
- I don’t know.

**Is cannabis more or less effective than your prescription medication for providing symptom relief?**

- Cannabis is much more effective than prescription medication.
- Cannabis is slightly more effective than prescription medication.
- Cannabis works about the same as prescription medication.
- Prescription medication is slightly more effective than cannabis.
- Prescription medication is much more effective than cannabis.
- I don’t know

**How are the side effects of the prescription medication compared to the side effects of cannabis?**

- The side effects of the prescription medication are much worse than those of cannabis.
- The side effects of the prescription medication are slightly worse than those of cannabis.
- There is no difference in side effects.
- The side effects of cannabis are slightly worse than those of the prescription medication.
- The side effects of cannabis are much worse than those of the prescription medication.
- I don’t know.

**Experience with healthcare**

Have you ever talked to a doctor about your use of cannabis for medicinal purposes? Yes/No

Have you ever asked a doctor to give you a prescription for medical cannabis? Yes/No

Have you ever received prescribed medical cannabis? Yes/No

Are you currently using prescribed medical cannabis? Yes/No

*If NO to item 2 (from the 4 items above), show this:*

**I have never asked a doctor for a medical cannabis prescription because:**

*Choose all that apply.*

- I did not know I could get medical cannabis on prescription.
- I do not think my doctor would prescribe medical cannabis to me.
- I do not think my doctor would know which type of cannabis to prescribe.
- I do not feel comfortable asking my doctor for a prescription for medical cannabis.
- I do not want my doctor to know I use cannabis for medicinal purposes.
- I do not want cannabis from a pharmacy.
- I do not need a doctor; I know which cannabis works best for me.
- Prescribed medical cannabis is too expensive.
- Other: ___________

*If YES to item 2 and NO to item 3 (from the 4 items above), show this:*

**My doctor did not give me a prescription for medical cannabis because:**

*Choose all that apply.*

- My doctor said my illness/condition does not quality for medical cannabis.
- My doctor did not know enough about medical cannabis to prescribe it to me.
- My doctor thinks cannabis is harmful.
- My doctor wanted me to try other medications first.
- My doctor does not believe that medical cannabis is effective.
- Other: ________

*If YES to item 3 and NO to item 4 (from the 4 items above), show this:*

**You indicated that you have used prescribed medical cannabis in the past but are no longer using it.**

What medical cannabis product did you use in the past? (Name, THC/CBD percentage) ___________

Why did you stop using prescribed medical cannabis? ____________

*If YES to item 4 (from the 4 items above), show this:*

**You indicated that you are currently using prescribed medical cannabis.**

What medical cannabis product do you use? (Name, THC/CBD percentage) ___________

Why do you also use non-prescribed cannabis for medicinal purposes? ____________

*Show these items to respondents who completed the last 2 items on ‘MC past’ or ‘MC current’:*

**To what extent do you agree with the following statements?**

***Non-prescribed cannabis is more effective for my symptoms than prescribed medical cannabis.***

- Strongly agree
- Agree
- They work equally well
- Disagree
- Strongly disagree

***Non-prescribed cannabis is more pleasant to use than prescribed medical cannabis (for example, the taste or smell is nicer).***

- Strongly agree
- Agree
- They are equally pleasant to use
- Disagree
- Strongly disagree

**Is there anything else you would like to share about your experience with prescribed medical cannabis?** ___

*(Non-mandatory item)*

*Show these items to everyone again.*

**Why do you use non-prescribed cannabis instead of prescribed medical cannabis?**

*Choose up to 3 reasons.*

- Non-prescribed cannabis is cheaper
- Non-prescribed cannabis is easier to obtain
- Non-prescribed cannabis is of better quality
- Pharmacies do not have the cannabis products I want
- Pharmacies do not have enough variety of cannabis products
- My doctor does not want to give me a prescription for medical cannabis
- I do not want my doctor to know I use cannabis for medicinal purposes
- I want to decide myself how I use cannabis for medicinal purposes
- I did not know I could get medical cannabis on prescription
- Other: _____

**Have you heard negative things about the prescribed medical cannabis?**

If yes, write down what you heard: ______

*(Non-mandatory item)*

**What type of cannabis do you prefer to use?**

**These are the things I consider important when choosing the cannabis I use for medicinal purposes.**

*Choose up to five.*

- Indica-dominant
- Sativa-dominant
- Indica/Sativa blend
- High THC content
- Low THC content
- High CBD content
- THC:CBD balanced mix
- The terpenoid profile
- How the flower smells
- How the flower looks
- Name (e.g. Kush, Haze)
- Taste
- Organically grown (without chemical pesticides)
- Not irradiated
- I rely on what others tell me (e.g. coffeeshop staff, friends, Internet)
- Other: _____

**Costs and stress**

**How much do you spend on the cannabis that you use for medicinal purposes per MONTH?**

- _____ Euros per month (fill in the amount here)
- I do not pay for my cannabis.
- Prefer not to say.

**Do you have financial worries because you use cannabis as medicine?**

- Not at all
- A little
- Somewhat
- Very much

**Do you ever worry about your use of cannabis as medicine in relation to the following:**

*Answer each item.*

The illegal status of cannabis Yes/No

Stigma Yes/No

Health issues (e.g., lungs) Yes/No

Contamination (e.g., pesticides) Yes/No

Uncertainty about whether my cannabis product is always available Yes/No

Risk of addiction Yes/No

**Would you like to share anything else about your medicinal use of cannabis? Or would you like to say something about this survey?** _______

*(Non-mandatory item)*

**Thank you very much for completing this survey!**

In a moment, you can take part in a lottery for a chance to win 200 Euro.
You are first given the opportunity to sign up for follow-up studies for this project. Participation is voluntary.

If you want to take part in one or both follow-up studies, we will ask for your email address. The **privacy statement** explains how we handle your information and what your rights are.

Do you know someone who uses cannabis as medicine? Please share this link with them: <https://trimbos.nl/medusa/>

The more people share their experiences, the better!

**Follow-up Study 1: An Interview.**

We would like to know from you:

Why do you not obtain medical cannabis on prescription from a doctor?

What do you like or not like about the way you currently obtain your cannabis?

The interview lasts 45-60 minutes and takes place online. Participants receive **40 Euro as compensation.**

More information about the study, how we handle your data, and your rights can be found in the information letter and privacy statement.

**Are you interested in participating?**

- Yes, I would like more information about the study. My email address is: ______
- No

**Follow-up Study 2: Sending Your Cannabis.**

We would like to know how much THC and CBD is in the weed/hash you use for your symptoms.

This helps us understand which types of cannabis products patients prefer.

To measure THC and CBD accurately, we need 1.5 grams of your weed or hash.

Participants receive **40 Euro as compensation.**

More information about the study, how we handle your data, and your rights can be found in the information letter and privacy statement.

**Are you interested in participating?**

- Yes, I would like more information about the study. My email address is: ______
- No

You can now enter a lottery for a chance to win one of 10 prizes of 200 Euro.

Click **THIS LINK** to participate. You will be taken to a page where you can enter your email address.

If you do not want to join the lottery, click **Next** to finish the survey.

**Enter your email address to join the lottery:**

Email: ______

We will notify you by email within 4 months if you win.

**Thank you very much for your participation in this study!**
